# In-depth meta-analysis: unilateral PKP demonstrates significant advantages in treating osteoporotic vertebral compression fractures—an expanded RCT study with GRADE scoring

**DOI:** 10.3389/fsurg.2025.1591686

**Published:** 2025-05-16

**Authors:** Lin Xiao, Wei Li, Jingxin Yan, Lei Luo, Ting Li

**Affiliations:** ^1^ Department of Orthopedics, The Sixth People’s Hospital of Chengdu, Chengdu, China; ^2^ School of Medicine, South China University of Technology, Guangzhou, China; ^3^ Department of Orthopedics, No. 1 Orthopedics Hospital of Chengdu, Chengdu, China

**Keywords:** meta-analysis, osteoporotic vertebral compression fractures, RCTs, percutaneous kyphoplasty, unilateral and bilateral

## Abstract

**Background:**

Percutaneous kyphoplasty (PKP) has become a mainstream intervention for osteoporotic vertebral compression fractures (OVCFs). While existing systematic reviews comparing unilateral and bilateral PKP approaches provide preliminary insights, they are limited by methodological inconsistencies and inconclusive evidence regarding comparative efficacy.

**Methods:**

We conducted a comprehensive systematic review of randomized controlled trials (RCTs) up to July 2024, searching major English databases (Cochrane Library, Embase, PubMed, Scopus, Web of Science) and Chinese databases (CNKI, VIP, and Wanfang).

**Results:**

The pooled analysis of 35 RCTs (*N* = 3,362) revealed no statistically significant differences between unilateral and bilateral PKP in long-term outcomes, including visual analog scale scores (*P* = 0.62), Oswestry Disability Index scores (*P* = 0.77), and Cobb angle correction (*P* = 0.64). However, unilateral PKP demonstrated significant perioperative advantages: shorter operative time (*P* < 0.00001), a lower dose of bone cement injection (*P* < 0.00001), and a reduced radiation dose (*P* < 0.00001). Furthermore, the study also found that unilateral PKP had a lower rate of bone cement leakage (*P* < 0.0001) and a reduced overall complication rate (*P* < 0.0001) compared to bilateral PKP.

**Conclusion:**

Unilateral PKP offers advantages over bilateral PKP, including shorter operation time, lower polymethylmethacrylate injection dose, reduced radiation exposure, lower bone cement leakage, and fewer complications. Therefore, unilateral PKP may be a preferable option for patients with OVCF, providing similar clinical outcomes with reduced procedural risks and resource requirements.

## Introduction

Osteoporotic vertebral compression fractures (OVCFs) represent a growing global health burden, with osteoporosis affecting over 200 million individuals worldwide and contributing to approximately 700,000 vertebral fractures annually in the United States alone (1, 2). This condition, characterized by diminished bone mineral density (BMD) and disrupted trabecular architecture, disproportionately impacts postmenopausal women due to estrogen deficiency but increasingly affects aging males, particularly those undergoing androgen deprivation therapy (2–4). Key modifiable risk factors include inadequate calcium/vitamin D intake, a sedentary lifestyle, and secondary endocrine dysregulation (5). OVCFs frequently occur spontaneously or following minimal trauma, manifesting as acute pain, progressive kyphotic deformity, and functional decline that collectively impair quality of life and independence (6). The socioeconomic consequences are substantial, with U.S. healthcare costs exceeding $18 billion annually for osteoporosis-related fractures.

Percutaneous kyphoplasty (PKP) has revolutionized OVCF management through minimally invasive vertebral augmentation using polymethylmethacrylate (PMMA) cement (7, 8). Despite its widespread use, the optimal approach, i.e., unilateral vs. bilateral PKP, remains a subject of debate among clinicians. Unilateral PKP involves the injection of bone cement through a single access point, while bilateral PKP requires two access points, one on each side of the vertebra (9). Proponents of bilateral PKP argue that it offers better vertebral body symmetry and improved cement distribution, potentially leading to superior clinical outcomes (10). Conversely, advocates of unilateral PKP highlight its simplicity, reduced procedural time, and lower risk of complications, such as bone cement leakage and radiation exposure (10, 11).

The current clinical guidelines lack consensus on the optimal surgical approach (unilateral vs. bilateral PKP) due to insufficient high-quality comparative evidence. This review intends to provide clinicians with evidence-based selection criteria to prioritize unilateral PKP for frail patients or resource-limited settings and reserve bilateral approaches for cases requiring enhanced vertebral stabilization. By establishing evidence-based selection criteria, this work advances standardized yet personalized decision-making in OVCF management, ultimately improving clinical outcomes and healthcare efficiency.

## Materials and methods

### Study design

This PRISMA-guided meta-analysis systematically compares the efficacy and safety profiles of unilateral vs. bilateral PKP in managing OVCFs with rigorous adherence to the Preferred Reporting Items for Systematic Reviews and Meta-Analyses guidelines (12).

### Literature retrieval strategy

A comprehensive search of electronic databases, including English databases (Cochrane Library, Embase, PubMed, Scopus, and Web of Science) and Chinese databases [China National Knowledge Infrastructure (CNKI), Chongqing VIP (VIP), and Wan Fang], was performed up to July 2024. We manually searched the bibliographies of randomized controlled trials (RCTs) comparing unilateral and bilateral PKP for OVCFs. Our search strategy incorporated both subject headings and free-text keywords: [(vertebral compression fracture OR osteoporosis) AND (percutaneous kyphoplasty OR unilateral OR bilateral)] AND (RCT). This literature retrieval strategy is detailed in Supplementary File 1.

### Inclusion and exclusion criteria

#### Inclusion criteria

To compare the advantages and disadvantages and the clinical outcomes of unilateral and bilateral PKP, studies were included based on the following PICOS criteria:

Population (P): Patients with OVCFs.

Intervention (I) vs. Control (C): Unilateral vs. bilateral PKP.

Outcomes (O): Operation time, bone cement injection dose, x-ray radiation dose, Cobb angle, visual analog scale (VAS) and Oswestry Disability Index (ODI) at preoperative and postoperative follow-up time points, bone cement leakage, and overall complication rate.

Study Design (S): Only RCTs.

#### Exclusion criteria

Studies were excluded based on the following criteria:

P: Non-osteoporotic vertebral fractures (e.g., traumatic, neoplastic, or infectious fractures). Patients with non-vertebral fragility fractures fulfilling WHO osteoporosis diagnostic criteria (T-score ≤ −2.5) (e.g., hip or wrist fractures).

I vs. C: Studies comparing PKP with non-PKP techniques (e.g., vertebroplasty, conservative therapy). Hybrid approaches (e.g., unilateral PKP + contralateral vertebroplasty or pedicle screw placement).

O: Studies lacking quantitative data on prespecified outcomes (operation time, cement volume, VAS/ODI, Cobb angle, complications).

S: Non-RCTs (e.g., cohort studies, case series).

Additional exclusions: Duplicate publications or overlapping datasets. Studies with <12 months of follow-up for primary outcomes. Patients with spinal comorbidities (e.g., degenerative stenosis, prior fusion surgery).

### Data extraction

Data were extracted jointly by two reviewers and screened and sorted using the Microsoft Word 2021 table tool in accordance with the Cochrane Collaboration guidelines for systematic reviews. Any disagreements between the reviewers were resolved by consultation with a third reviewer. All studies included in the study were collected based on outcome metrics (authorship, year, participants, intervention treatment, control treatment, vertebral fractures, and clinical outcomes). When information was lacking, we tried to reach out to the primary author via email to obtain clarification or to exclude the study.

### Risk of bias assessment

The assessment of bias in the included studies was performed utilizing the Cochrane tool (https://methods.cochrane.org/risk-bias-2). The tool assesses the following aspects of bias (13). (1) Random sequence generation: Assessed whether randomization methods (e.g., computer-generated numbers, block randomization) were explicitly reported. For example, studies using “computer-generated numbers” were classified as low risk; those stating only “randomized” without details were classified as unclear risk. (2) Allocation concealment: Evaluated safeguards against selection bias (e.g., centralized allocation vs. open randomization lists). For example, studies not using “sealed opaque envelopes” were classified as high risk. (3) Blinding: A study design in which both the investigators and participants remain unaware of group assignments is termed double-blind. For example, an article that describes the utilization of a double-blind methodology is considered to be associated with a low risk of bias. (4) Incomplete outcome data: A threshold of >20% loss to follow-up without intention-to-treat analysis was classified as high risk. (5) Selective reporting: Cross-checked outcomes against trial registrations and protocols. The evaluation of bias was performed by two independent researchers, and the overall quality assessment was carried out by the same two reviewers. RevMan 5.4 was used to construct a risk bias map.

### Statistical analysis

The meta-analysis was conducted for each outcome using RevMan 5.4 software. For the incidence of cement leakage and overall complication, odds ratios (ORs) and 95% confidence intervals (95% CIs) were calculated using the dichotomous variable method. The standardized mean differences (SMD) and mean differences (MD) were calculated using the continuous variable method. To evaluate the heterogeneity of the included studies, we applied the Chi-square test. A lack of heterogeneity was indicated by *P* ≥ 0.1 and *I*^2^ ≤ 50%, which led to the use of a fixed-effect model. If *I*^2^ < 50% or *P* < 0.1, a random-effects model was used. In addition, we evaluated publication bias by generating funnel plots corresponding to each category of failure mode.

We strictly adhered to the Grading of Recommendations, Assessment, Development, and Evaluations (GRADE) guidelines for assessing risk ratios, applying a 1-point downgrade if the 95% confidence interval of the risk ratio crossed the null value. Additional downgrades for imprecision were applied to very small sample sizes in the pooled analyses: a “serious” quality downgrade was used for sample sizes with one study arm of <50 individuals and a “very serious” quality downgrade was used for total sample sizes ≤30 individuals. GRADE quality assessments were conducted by two independent reviewers, who resolved discrepancies through discussion and consensus.

## Results

### Search result

**Figure 1:**
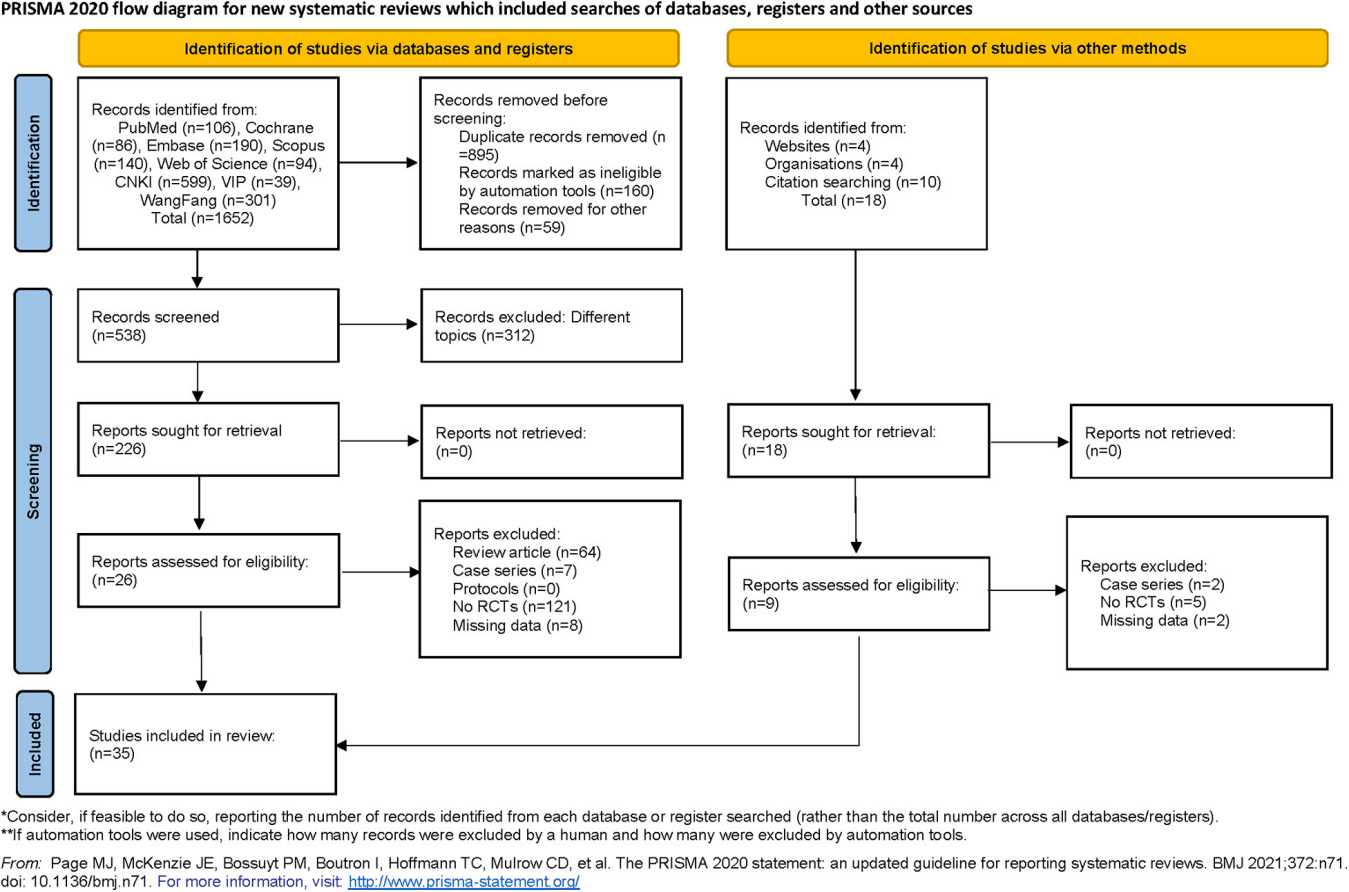
The flowchart of the study.

Our systematic search identified 1,670 potentially relevant articles published between 1990 and 2024. After removing 895 duplicates through automated and manual verification, two independent reviewers screened the titles/abstracts of 775 unique records. Subsequently, 312 articles were excluded due to non-target topics, 7 due to case report formats, and 64 due to review article types during full-text evaluation. Ultimately, 35 studies (17 English-language and 18 Chinese-language RCTs) met the predefined inclusion criteria and were included in the meta-analysis (14–48). Figure 1 shows the selection process for the relevant studies.

### Study characteristics

A total of 35 RCTs comparing unilateral vs. bilateral PKP for the treatment of OVCFs were retrieved and analyzed, involving a total of 3,362 participants. Participants' ages ranged from 62.13 to 82.5 years, and the sample sizes ranged from 22 to 383. The shortest follow-up time was 12 months, and the longest was 98 months. Two articles lacked participant age information, 15 articles did not specify the location of vertebral fractures, and 9 articles were missing data on study duration. The basic characteristics of the included studies are detailed in Table 1.

**Table 1:** Basic characteristics of the included studies.

Author	Year	Number of persons (I/C)	Age (I/C)	Intervention group	Control group	Vertebral fractures (I/C)	Study duration (months)	Outcome
Ceng YW	2013	12/14	NA	Unilateral bone cement	Bilateral bone cement	NA	6–12 (9)	②⑧
Chen CM	2010	33/25	67.73/68.52	Unilateral bone cement	Bilateral bone cement	NA	NA	①②⑧⑨
Chen CM	2014	20/19	69.43/68.66	Unilateral bone cement	Bilateral bone cement	NA	NA	①②⑨⑫⑬
Chen L	2011	24/25	70.4/72.4	Unilateral bone cement	Bilateral bone cement	Lumbar 26 (I)/23(C); thoracic 29 (I)/36(C)	31.8/35.2	④⑤⑥⑦⑫⑬
Cheng YH	2019	26/22	68.9/69.8	Unilateral bone cement	Bilateral bone cement	Lumbar 14 (I)/16(C); thoracic 16 (I)/16(C)	3	①②③⑧⑫⑬
Feng YH	2023	50/50	63.98/63.87	Unilateral bone cement	Bilateral bone cement	Lumbar 22 (I)/20(C); thoracic 28 (I)/30(C)	NA	①②③⑦⑧⑨⑫⑬
Geng ZH	2021	40/31	70.6/70.4	Unilateral bone cement	Bilateral bone cement	Lumbar 27 (I)/19(C); thoracic 13 (I)/12(C)	NA	①②⑦⑧⑨⑫⑬
Huang SC	2021	46/46	72.05/71.72	Unilateral bone cement	Bilateral bone cement	NA	NA	①②③④⑤⑧⑫⑬
Li L	2014	38/37	71.13/67.65	Unilateral bone cement	Bilateral bone cement	Lumbar 24 (I)/20(C); thoracic 18 (I)/20 (C)	24	①②③④⑤⑦⑧⑩⑪⑪⑫⑬
Li Q	2012	50/41	73.1/70.8	Unilateral bone cement	Bilateral bone cement	NA	12–36	①②③⑧
Liu CL	2015	48/50	70.14/70.52	Unilateral bone cement	Bilateral bone cement	NA	15.81/15.42	①②⑧⑨⑪⑫⑬
Liu MX	2018	42/43	67.7/70.5	Unilateral bone cement	Bilateral bone cement	NA	12	①②⑦⑧⑨⑪⑫⑬
Lu JH	2022	37/42	67.4/70.3	Unilateral bone cement	Bilateral bone cement	Lumbar 20 (I)/21(C); thoracic 17 (I)/21(C)	24	①②③④⑤⑥⑦⑪⑫⑬
Lu ZH	2022	175/208	72.3/74.1	Unilateral bone cement	Bilateral bone cement	Lumbar 79 (I)/93(C); thoracic 96 (I)/115(C)	28–98 (43.3)	①②③⑦⑧⑨⑩⑪⑫⑬
Mu ZZ	2022	80/73	62.13/63.51	Unilateral bone cement	Bilateral bone cement	Lumbar 44 (I)/43(C); thoracic 36 (I)/36(C)	29.92/30.28	⑪⑫⑬
Rebollede BJ	2013	23/21	78.7/79.3	Unilateral bone cement	Bilateral bone cement	Lumbar 7 (I)/2(C); thoracic 21 (I)/26(C)	NA	①②⑦⑫⑬
Shi X	2022	40/45	71.38/70.64	Unilateral bone cement	Bilateral bone cement	NA	NA	①②③④⑧⑨⑫⑬
Tan HT	2018	66/66	69.3/68.4	Unilateral bone cement	Bilateral bone cement	Lumbar 35 (I)/38(C); thoracic 44 (I)/48(C)	12	①③④⑦⑧⑫⑬
Tang J	2019	83/95	72.3/73.9	Unilateral bone cement	Bilateral bone cement	Lumbar 42 (I)/51(C); thoracic 41 (I)/44(C)	9.3/8.5	①②③④⑤⑦⑧⑨⑪⑬
Xiong XM	2019	38/25	69.7/69.4	Unilateral bone cement	Bilateral bone cement	Lumbar 26 (I)/14(C); thoracic 12 (I)/11(C)	12	①②③⑤⑦⑧⑨
Xu DL	2024	62/74	69.4/68.8	Unilateral bone cement	Bilateral bone cement	Lumbar 36 (I)/39(C); thoracic 26 (I)/35(C)	12	①②③⑦⑧⑨⑫⑬
Xue W	2017	38/38	67.89/69.37	Unilateral bone cement	Bilateral bone cement	Lumbar 23 (I)/22(C); thoracic 15 (I)/16(C)	12	①②⑤⑦⑧⑨
Yan L	2015	55/53	68.9	Unilateral bone cement	Bilateral bone cement	NA	12	①②⑦⑧⑫⑬
Yan L	2014	158/151	71.9/71.1	Unilateral bone cement	Bilateral bone cement	NA	12–28 (16.8)	①②⑦⑪⑫⑬
Yang AF	2018	45/46	75.2/76.1	Unilateral bone cement	Bilateral bone cement	Lumbar 20 (I)/21(C); thoracic 25 (I)/25(C)	6–12	①③⑦⑧⑨⑩⑪⑬
Yin F	2016	11/11	81.3/82.5	Unilateral bone cement	Bilateral bone cement	Thoracic 11 (I)/11(C)	13–35 (15.3)	①③⑦⑧⑫⑬
Yu Q	2020	16/16	68.74/70.91	Unilateral bone cement	Bilateral bone cement	NA	6	⑫⑬
Zhang L	2015	24/26	69.2/70.5	Unilateral bone cement	Bilateral bone cement	NA	24	①②③⑧⑨⑫⑬
Zhang LC	2023	36/35	72.69/71.86	Unilateral bone cement	Bilateral bone cement	Lumbar 31 (I)/30(C); thoracic 9 (I)/10(C)	NA	①②③⑧⑨
Zhang LG	2015	36/32	70/70.7	Unilateral bone cement	Bilateral bone cement	NA	12	⑧⑫⑬
Zhang YH	2020	32/28	NA	Unilateral bone cement	Bilateral bone cement	Lumbar 11 (I)/10(C); thoracic 25(I)/23(C)	6–13	①⑧⑫⑬
Zhang YT	2022	29/38	73.6/74.1	Unilateral bone cement	Bilateral bone cement	NA	14–27 (17.1)	①②⑧
Zhou MW	2013	30/37	67.1/67.1	Unilateral bone cement	Bilateral bone cement	NA	18–54 (28.6)	①②④⑤⑦⑧⑪⑫⑬
Zhou RL	2020	59/59	72.3/72.3	Unilateral bone cement	Bilateral bone cement	Lumbar 30 (I)/32(C); thoracic 16(I)/15 (C)	NA	①②③④⑤⑥⑦⑧⑨⑫⑬
Zhou X	2019	69/69	71.47/70.47	Unilateral bone cement	Bilateral bone cement	Lumbar 14 (I)/16(C); thoracic 12(I)/10(C)	12	①②③⑦⑩⑪⑫⑬

① Operation time (min); ② cement dose (ml); ③ radiation dose; ④ anterior vertebral height; ⑤ Middle vertebral height; ⑥ Posterior vertebral height; ⑦ Cobb angle (°); ⑧ VAS; ⑨ ODI; ⑩ hospital stays; ⑪ refracture; ⑫ cement leakage; ⑬ overall complication rate; NA, not available.

### Bias risk assessment

**Figure 2:**
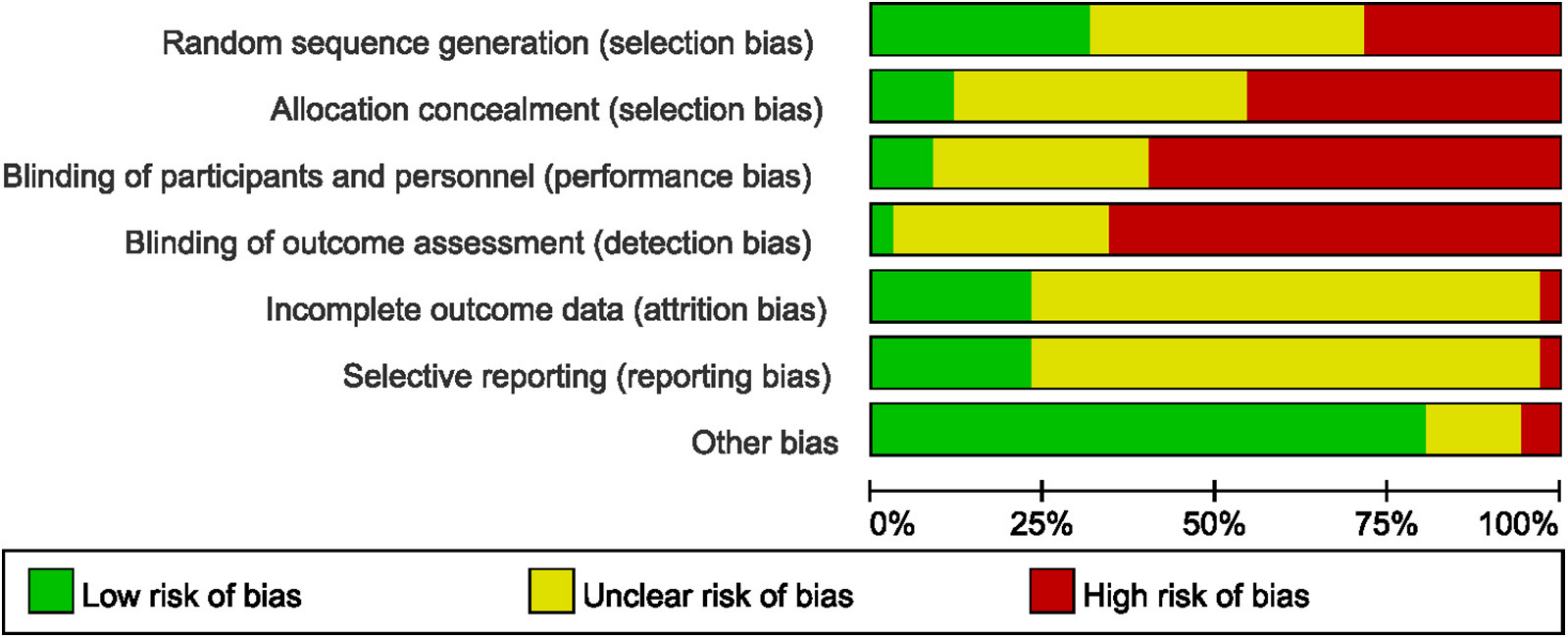
Results of quality assessment using the Cochrane Risk of Bias tool.

The bias risk assessment of the included trials based on Cochrane criteria is summarized in Figure 2. The results for each quality item are presented as percentages across studies. Ten articles did not report RCT design details, 14 articles had ambiguous random sequence generation, and 11 studies explicitly stated RCT design. Furthermore, 16 articles did not report details of allocation concealment, 15 articles provided unclear descriptions of allocation concealment, and 4 articles explicitly detailed the specifics of allocation concealment. Moreover, 21 articles did not report details of the blinding method, 11 articles provided unclear descriptions of the blinding, and 3 articles explicitly detailed the specifics of the blinding method. The quality assessment at the outcome level, conducted using the GRADE methodology, is summarized in Table 2. The overall evidence quality, evaluated according to GRADE criteria, was determined to be moderate to very low.

**Table 2.** GRADE assessment of clinical outcomes.

Certainty assessment							No. of patients		Effect		Certainty	Importance
No. of studies	Study design	Risk of bias	Inconsistency	Indirectness	Imprecision	Other considerations	Unilateral PKP	Bilateral PKP	Relative (95% CI)	Absolute (95% CI)		
OT (*N* = 30)	RCTs	Serious^a^	Very serious^c^	Not serious	Very Serious ^e^	No	1,530	1,556	MD −15.09 (−17.72 to −12.46)		⊕◯◯◯ Very low	Important
Bone cement dose (*N* = 27)	RCTs	Serious^a^	Very serious^c^	Not serious	No	No	1,355	1,402	MD −1.34 (−1.76 to −0.93)		⊕◯◯◯ Very low	Important
Radiation dose (*N* = 18)	RCTs	Serious^a^	Very serious^c^	Not serious	Very Serious ^e^	No	955	997	SMD −2.14 (−2.62 to −1.67)		⊕◯◯◯ Very low	Important
Cobb angle (*N* = 20)	RCTs	Serious^a^	Serious^b^	Not serious	No	No	1,902	1,953	MD 0.05 (−0.17 to 0.28)		⊕⊕◯◯ Low	Important
VAS (*N* = 27)	RCTs	Serious^a^	Not serious	Not serious	Not serious	No	2,559	2,598	MD 0.01 (−0.03 to 0.05)		⊕⊕⊕◯ Moderate	Important
ODI (*N* = 16)	RCTs	Serious^a^	Serious^b^	Not serious	Not serious	No	1,700	1,745	MD −0.06 (−0.44 to 0.32)		⊕⊕◯◯ Low	Important
Bone cement leakage (*N* = 26)	RCTs	Serious^a^	No serious	Not serious	Not serious	No	165/1,330 (12.4%)	236/1,355 (17.4%)	OR 0.64 (0.51 to 0.80)		⊕⊕⊕◯ Moderate	Important
Overall complication (*N* = 28)	RCTs	Serious^a^	Not serious	Not serious	Not serious	No	243/1,548 (15.7%)	334/1,516 (22.0%)	OR 0.67 (0.56 to 0.81)		⊕⊕⊕◯ Moderate	Important
Anterior vertebral height (*N* = 9)	RCTs	Serious^a^	Not serious	Not serious	Serious^d^	No	422	453	SMD −0.04 (−0.17 to 0.1)		⊕⊕◯◯ Low	Not important
Middle vertebral height (*N* = 9)	RCTs	Serious^a^	Not serious	Not serious	Serious^d^	No	392	405	SMD −0.05 (−0.19 to 0.09)		⊕⊕◯◯ Low	Not important
Posterior vertebral height (*N* = 3)	RCTs	Serious^a^	Not serious	Not serious	Serious^d^	No	120	126	SMD 0.04 (−0.21 to 0.29)		⊕⊕◯◯ Low	Not important
6 months follow-up height (*N* = 5)	RCTs	Serious^a^	Not serious	Not serious	Serious^d^	No	440	460	SMD −0.14 (−0.27 to 0.00)		⊕⊕◯◯ Low	Not important
12 months follow-up height (*N* = 3)	RCTs	Serious^a^	Not serious	Not serious	Not serious	No	142	129	MD 0.07 (−0.33 to 0.47)		⊕⊕⊕◯ Moderate	Not important
Cobb angle improvement (*N* = 3)	RCTs	Serious^a^	Not serious	Not serious	Very Serious ^e^	No	144	137	SMD −0.03 (−0.35 to 0.29)		⊕◯◯◯ Very low	Not important
Hospital days (*N* = 4)	RCTs	Serious^a^	Not serious	Not serious	Not serious	No	327	360	MD −0.16 (−0.57 to 0.26)		⊕⊕⊕◯ Moderate	Not important
Refracture (*N* = 11)	RCTs	Serious^a^	Not serious	Not serious	Not serious	No	73/802 (9.1%)	75/846 (8.9%)	OR 1.00 (0.71 to 1.40)		⊕⊕⊕◯ Moderate	Important
Postoperative pain (*N* = 3)	RCTs	Serious^a^	Not serious	Not serious	Not serious	No	17/208 (8.2%)	25/211 (11.8%)	OR 0.60 (0.31 to 1.17)		⊕⊕⊕◯ Moderate	Not important

CI, confidence interval; OR, odds ratio; SMD, standardized mean difference; MD, mean difference.

^a^ >50% of trials received a “high” risk of bias rating (≥1 out of six dimensions in the Cochrane Risk of Bias tool).

^b^ *I*^2^ between >50% and ≤75% in either direction.

^c^ *I*^2^ > 75% in either direction.

^d^ 95% CI of an SMD extends between >0.2 and ≤0.5 points in either direction, 95% CI of an MD extends between >2.0 and ≤5.0 points in either direction.

^e^ 95% CI of an SMD extends >0.5 points in either direction, 95% CI of an MD extends >5.0 points in either direction.

### Primary meta-analysis results

#### Operation time

**Figure 3:**
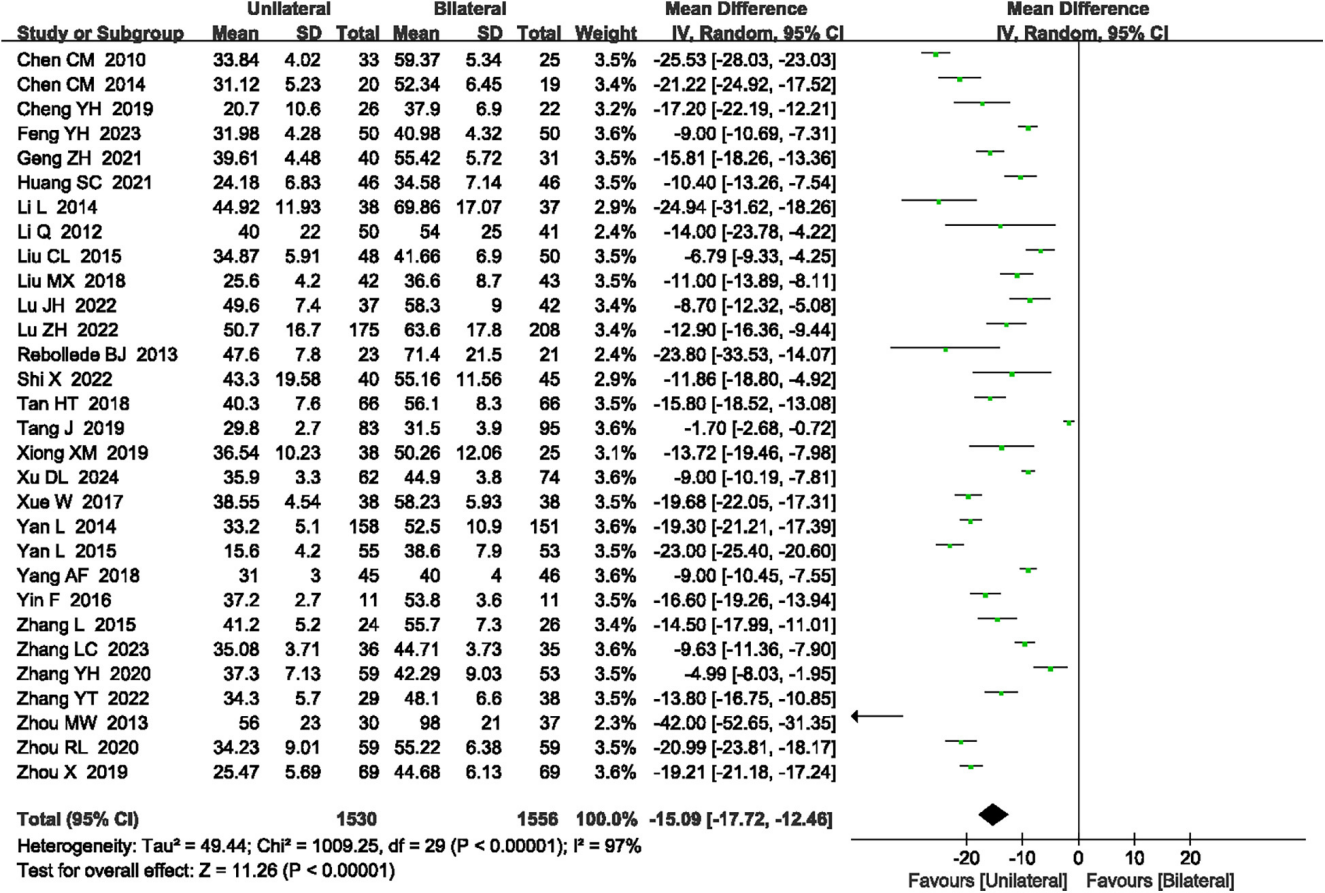
A forest plot showing the operation time.

A total of 30 articles (15, 16, 18–27, 29–39, 41, 42, 44–48) reported the operation time with high heterogeneity (*P* < 0.00001, *I*^2^ = 97%), prompting the use of a random-effects model. The results revealed that unilateral PKP had shorter operation times compared to bilateral PKP (SMD = −15.09, 95% CI: −17.72 to −12.46, *P* < 0.00001; Figure 3). A sensitivity analysis was conducted to explore potential sources of heterogeneity, but no specific source was identified. The outcome quality level for operation time, as assessed by GRADE, was “very low.”

#### Bone cement dose

**Figure 4:**
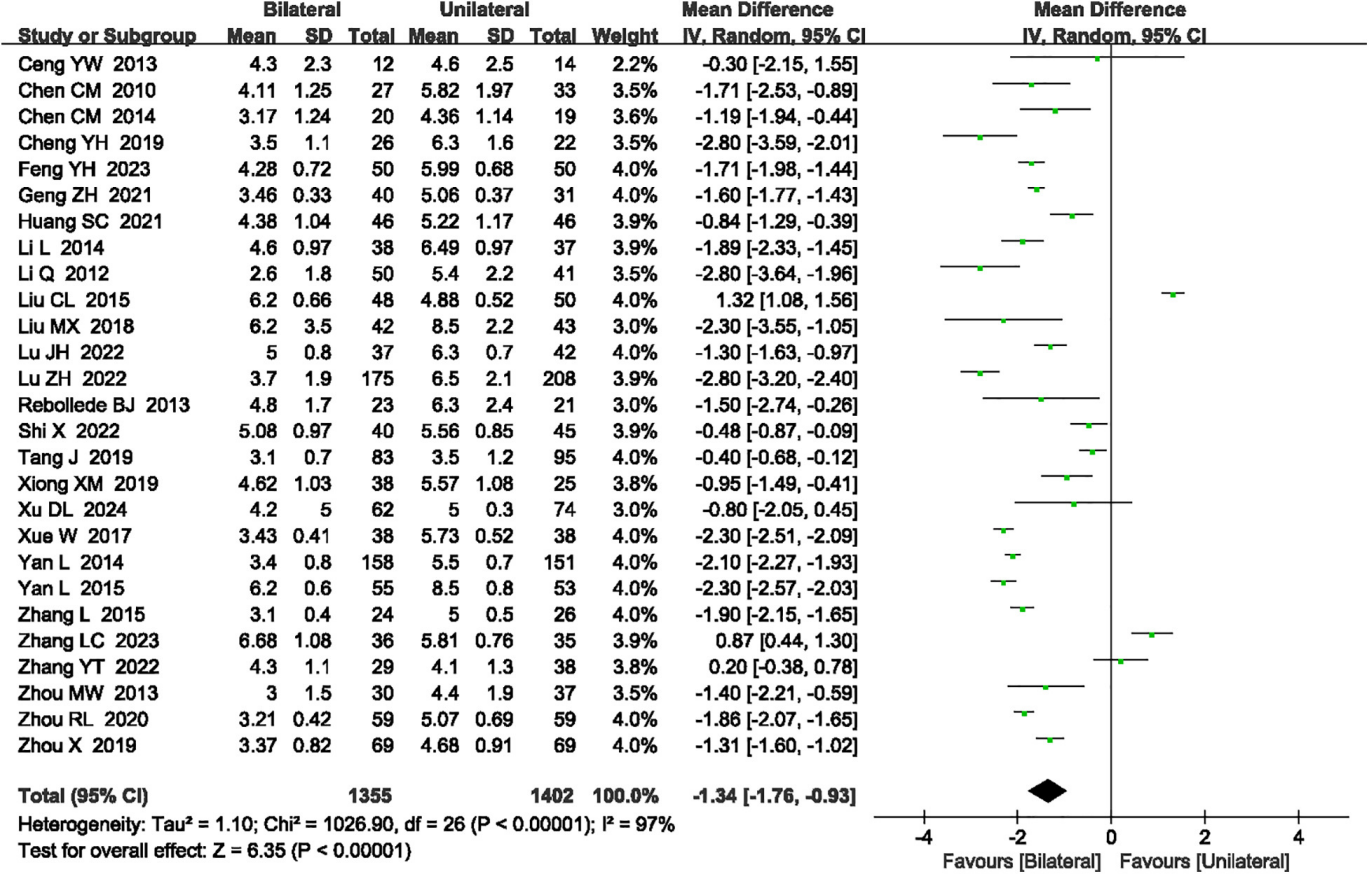
A forest plot showing the bone cement dose.

A total of 27 articles (14–16, 18–27, 29, 30, 32–37, 41, 42, 44, 46–48) reported the bone cement dose. Significant heterogeneity was detected (*P* < 0.00001, *I*^2^ = 97%), necessitating the use of a random-effects model. The analysis indicated that unilateral PKP had a lower bone cement dose compared to bilateral PKP (SMD = −1.34, 95% CI: −1.76 to −0.93, *P* < 0.00001; Figure 4). A sensitivity analysis was performed to identify potential sources of heterogeneity, but no specific source was found. The outcome quality level for bone cement dose, as assessed by GRADE, was “very low.”

#### Radiation dose

**Figure 5:**
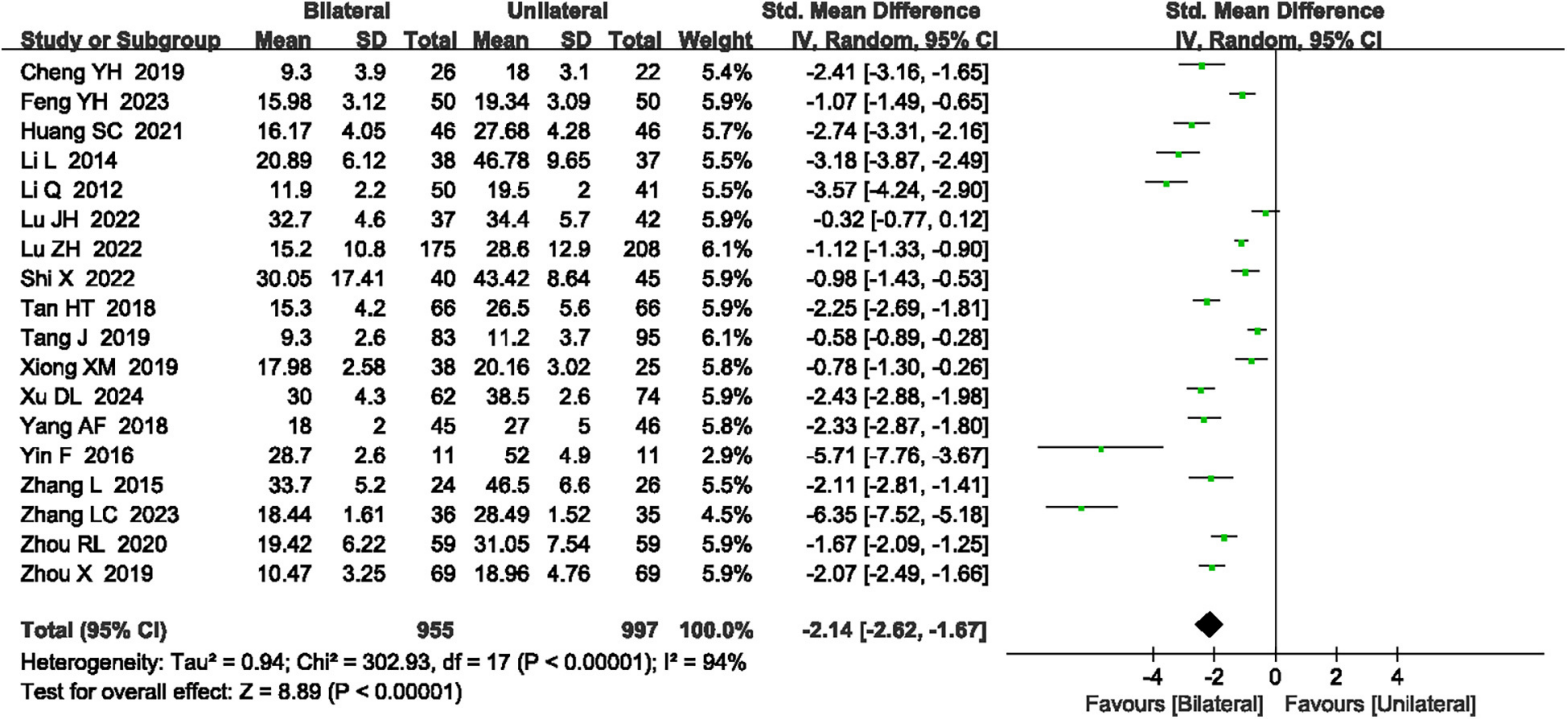
A forest plot showing the radiation dose.

A total of 18 articles (18, 19, 21–23, 26, 27, 30–34, 38, 39, 41, 42, 47, 48) reported the radiation dose. High heterogeneity was observed (*P* < 0.00001, *I*^2^ = 94%), requiring a random-effects model. The meta-analysis indicated that unilateral PKP had a lower radiation dose compared to bilateral PKP (SMD = −2.14, 95% CI: −2.62 to −1.67, *P* < 0.00001; Figure 5). A sensitivity analysis was conducted to explore potential sources of heterogeneity, but no specific sources were identified. The outcome quality level for radiation dose, as assessed by GRADE, was “very low.”

#### Cobb angle

**Figure 6:**
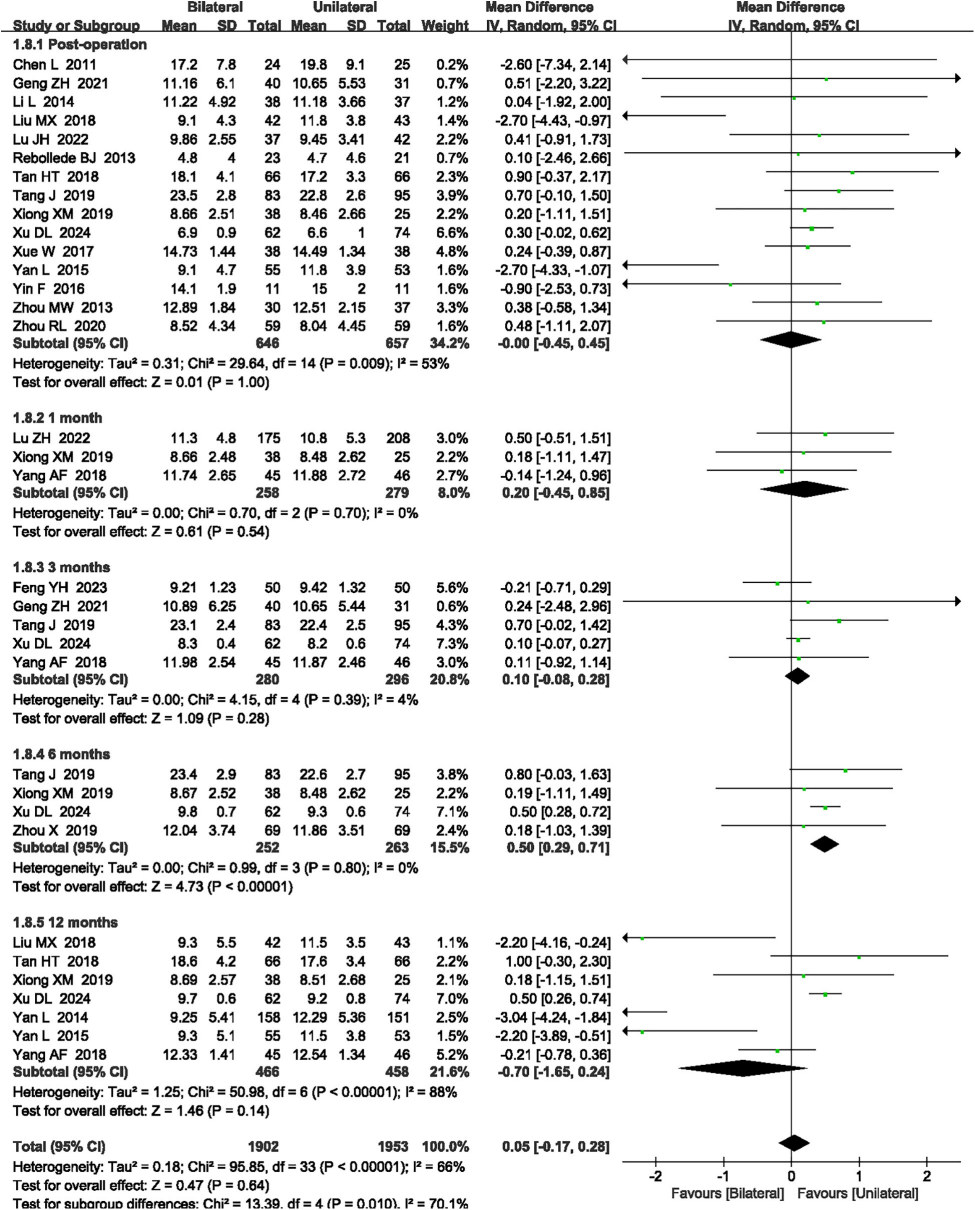
A forest plot showing the Cobb angle.

A total of 20 articles (17, 19, 20, 22, 25–27, 29, 31–39, 46–48) reported the Cobb angle at post-operation and 1, 3, 6, and 12 months after surgery. High heterogeneity was observed (*P* < 0.00001, *I*^2^ = 66%), so a random-effects model was applied. Although the meta-analysis showed that unilateral PKP had a larger Cobb angle compared to bilateral PKP at 6 months after surgery (MD = 0.50, 95% CI: 0.29–0.71, *P* < 0.00001, Figure 6), there was no significant difference in post-operation, and 1, 3, and 12 months after surgery between unilateral PKP and bilateral PKP (*P* = 1.00, *P* = 0.54, *P* = 0.28, and *P* = 0.14, respectively; Figure 6). The outcome quality level for Cobb angle, as assessed by GRADE, was “low.”

#### VAS scores

**Figure 7:**
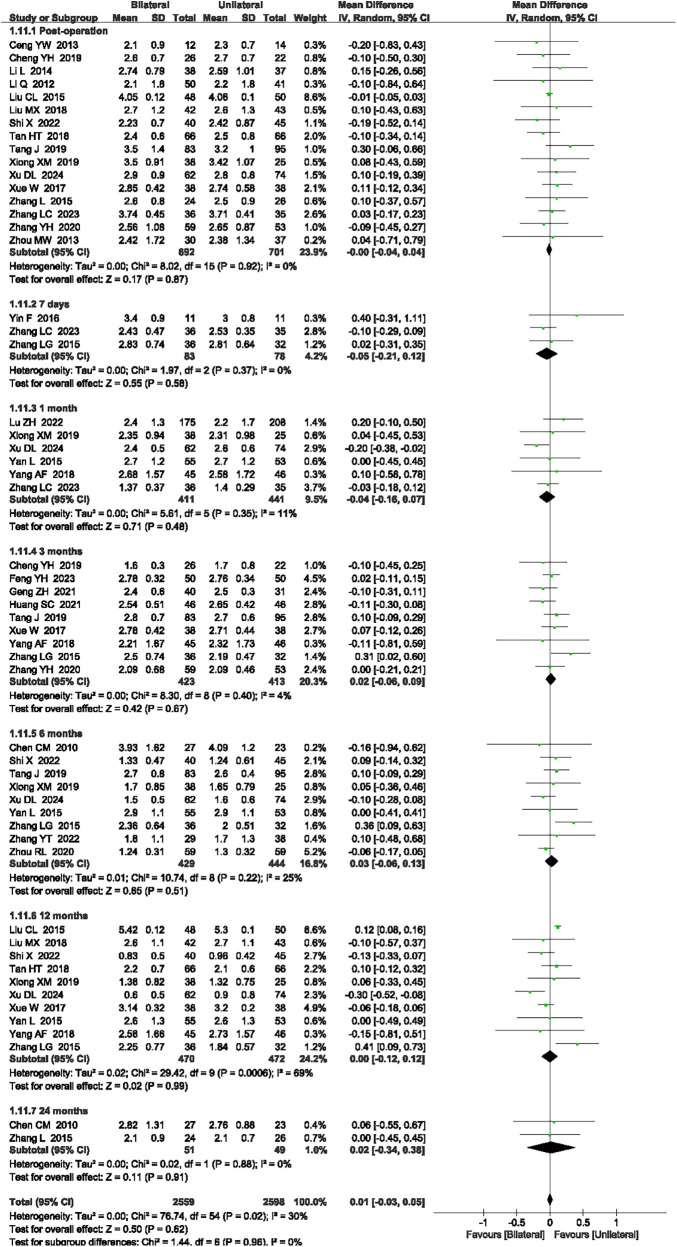
A forest plot showing the VAS scores.

A total of 27 articles (14, 16, 18–25, 27, 30–36, 38, 39, 41–47) reported the VAS scores at post-operation, and 7 days, 1 month, 3 months, 6 months, 12 months, and 24 months after surgery. The heterogeneity was 30% (*P* = 0.02, *I*^2^ = 30%), necessitating the use of a random-effects model. There was no significant difference post-operation and 7 days, 1 month, 3 months, 12 months, and 24 months after surgery between unilateral PKP and bilateral PKP for VAS scores (*P* = 0.87, *P* = 0.58, *P* = 0.48, *P* = 0.67, *P* = 51, *P* = 0.99, and *P* = 0.91, respectively, Figure 7). The outcome quality level for VAS scores, as assessed by GRADE, was “moderate.”

#### ODI scores

**Figure 8:**
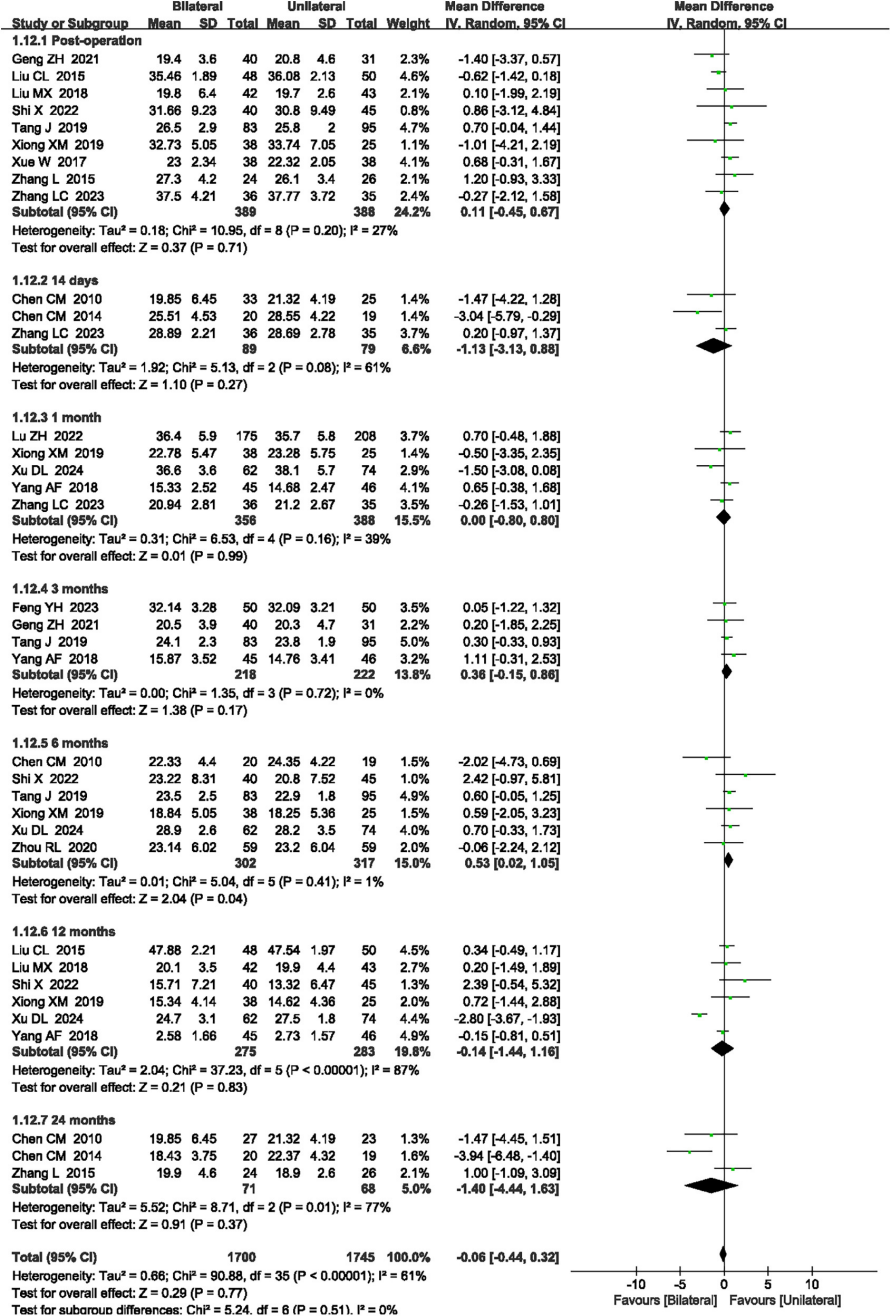
A forest plot showing the ODI scores.

A total of 16 articles (15, 16, 19, 20, 24, 25, 27, 30, 32–35, 38, 41, 42, 47) reported the ODI scores at post-operation and 14 days, 1 month, 3 months, 6 months, 12 months, and 24 months after surgery. High heterogeneity was observed (*P* < 0.00001, *I*^2^ = 61%), so a random-effects model was applied. Although the meta-analysis showed that unilateral PKP had a higher ODI score compared to bilateral PKP at 6 months after surgery (MD = 0.53, 95% CI: 0.02–1.05, *P* = 0.04, Figure 8), there was no significant difference post-operation and 14 days, 1 month, 3 months, 12 months, and 24 months after surgery between unilateral PKP and bilateral PKP (*P* = 0.71, *P* = 0.27, *P* = 0.99, *P* = 0.17, *P* = 0.83, and *P* = 0.37, respectively, Figure 8). The outcome quality level for ODI scores, as assessed by GRADE, was “low.”

#### Bone cement leakage

**Figure 9:**
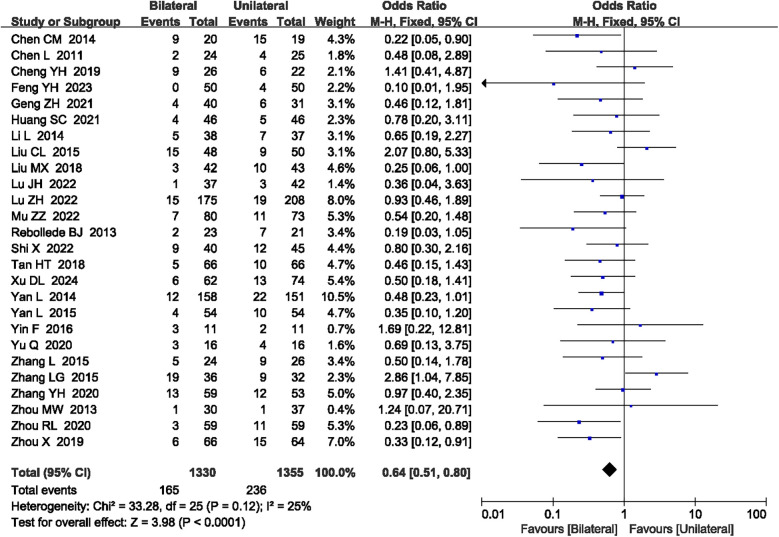
A forest plot showing the bone cement leakage.

A total of 26 articles (15, 17–22, 24–31, 34, 36, 37, 39–41, 43, 45–48) reported bone cement leakage. No heterogeneity was observed (*P* = 0.12, *I*^2^ = 25%), so a fixed-effect model was used. The meta-analysis showed that unilateral PKP had a lower bone cement leakage rate compared to bilateral PKP (OR = 0.64, 95% CI: 0.51–0.80, *P* < 0.0001; Figure 9). The outcome quality level for bone cement leakage, as assessed by GRADE, was “moderate.”

#### Overall complication rate

**Figure 10:**
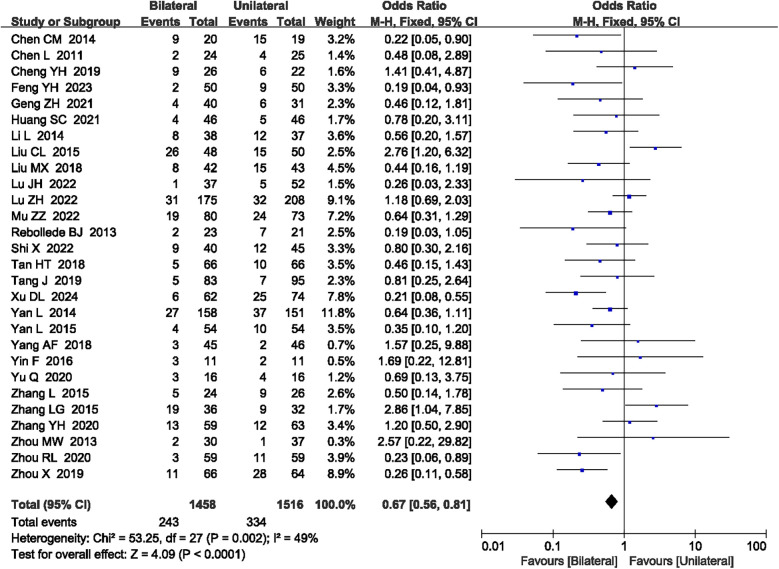
A forest plot showing the overall complication rate.

A total of 28 articles (15, 17–22, 24–32, 34, 36–41, 43, 45–48) reported the overall complication rate. No heterogeneity was observed (*P* = 0.002, *I*^2^ = 49%), so a fixed-effect model was used. The meta-analysis showed that unilateral PKP had a lower overall complication rate compared to bilateral PKP (OR = 0.67, 95% CI: 0.56–0.81, *P* < 0.0001; Figure 10). The outcome quality level for overall complication rate, as assessed by GRADE, was “moderate.”

### Secondary meta-analysis results

#### Vertebral height

A total of nine articles (17, 21, 22, 26, 30–32, 46, 47) reported the anterior vertebral height, nine articles (17, 21, 22, 26, 32, 33, 35, 46, 47) reported the mid-vertebral height, and three articles (17, 26, 47) reported the posterior vertebral height. These results did not have significant heterogeneity (*P* = 0.66, *I*^2^ = 0%; *P* = 0.59, *I*^2^ = 0%; *P* = 0.83, *I*^2^ = 0%, respectively), so fixed-effect model was used. There was no significant difference in anterior vertebral height (SMD = −0.04, 95% CI: −0.17 to 0.10, *P* = 0.59; see Supplementary File 2, Figure S1), mid-vertebral height (SMD = −0.05, 95% CI: −0.19 to 0.09, *P* = 0.51; see Supplementary File 2, Figure S2), and posterior vertebral height (SMD = 0.04, 95% CI: −0.21–0.29, *P* = 0.74; see Supplementary File 2, Figure S3) between unilateral PKP and bilateral PKP. The outcome quality level for vertebral height, as assessed by GRADE, was “low.”

#### Follow-up of vertebral height

A total of five articles (32, 33, 44, 46, 48) reported vertebral height at a 6-month follow-up, including anterior vertebral height and mid-vertebral height. No significant heterogeneity was observed (*P* = 0.59, *I*^2^ = 0%; *P* = 0.17, *I*^2^ = 41%, respectively), so a fixed-effect model was applied. There was no significant difference at a 6-month follow-up, including anterior vertebral height (SMD = −0.15, 95% CI: −0.34 to 0.03, *P* = 0.11; see Supplementary File 2, Figure S4) and mid-vertebral height (SMD = −0.12, 95% CI: −0.31 to 0.07, *P* = 0.22; see Supplementary File 2, Figure S4) between unilateral PKP and bilateral PKP. The outcome quality level for vertebral height at a 6-month follow-up, as assessed by GRADE, was “low.”

A total of three articles (31, 33, 35) reported vertebral height at a 12-month follow-up. No significant heterogeneity was observed (*P* = 0.21, *I*^2^ = 36%), so a fixed-effect model was applied. There was no significant difference at a 12-month follow-up between unilateral PKP and bilateral PKP (MD = 0.07, 95% CI: −0.33 to 0.47, *P* = 0.73; see Supplementary File 2, Figure S5). The outcome quality level for vertebral height at a 12-month follow-up, as assessed by GRADE, was “moderate.”

#### Cobb angle improvement

A total of three articles (21, 23, 24) reported Cobb angle improvement. No significant heterogeneity was observed (*P* = 0.16, *I*^2^ = 46%). There was no significant difference in Cobb angle improvement between unilateral PKP and bilateral PKP (SMD = −0.03, 95% CI: −0.35 to 0.29, *P* = 0.86; see Supplementary File 2, Figure S6). The outcome quality level for Cobb angle improvement, as assessed by GRADE, was “very low.”

#### Hospital stays

A total of four articles (22, 27, 38, 48) reported hospital stays. No significant heterogeneity was observed (*P* = 0.78, *I*^2^ = 0%). There was no significant difference in hospital stays between unilateral PKP and bilateral PKP (MD = −0.16, 95% CI: −0.57 to 0.26, *P* = 0.46; see Supplementary File 2, Figure S7). The outcome quality level for hospital stays, as assessed by GRADE, was “moderate.”

#### Refracture rate

A total of 11 articles (22, 24–28, 32, 37, 38, 46, 48) reported a refracture rate. No significant heterogeneity was observed (*P* = 0.71, *I*^2^ = 0%). There was no significant difference in refracture rate between unilateral PKP and bilateral PKP (OR = 1.00, 95% CI: 0.71–1.40, *P* = 1.00; see Supplementary File 2: Figure S8). The outcome quality level for refracture rate, as assessed by GRADE, was “moderate.”

#### Postoperative pain rate

A total of three articles (28, 34, 48) reported a postoperative pain rate. No significant heterogeneity was observed (*P* = 0.36, *I*^2^ = 1%). There was no significant difference in postoperative pain rate between unilateral PKP and bilateral PKP (OR = 0.60, 95% CI: 0.31–1.17, *P* = 0.13; see Supplementary File 2, Figure S9). The outcome quality level for postoperative pain rate, as assessed by GRADE, was “moderate.”

### Publication bias

**Figure 11:**
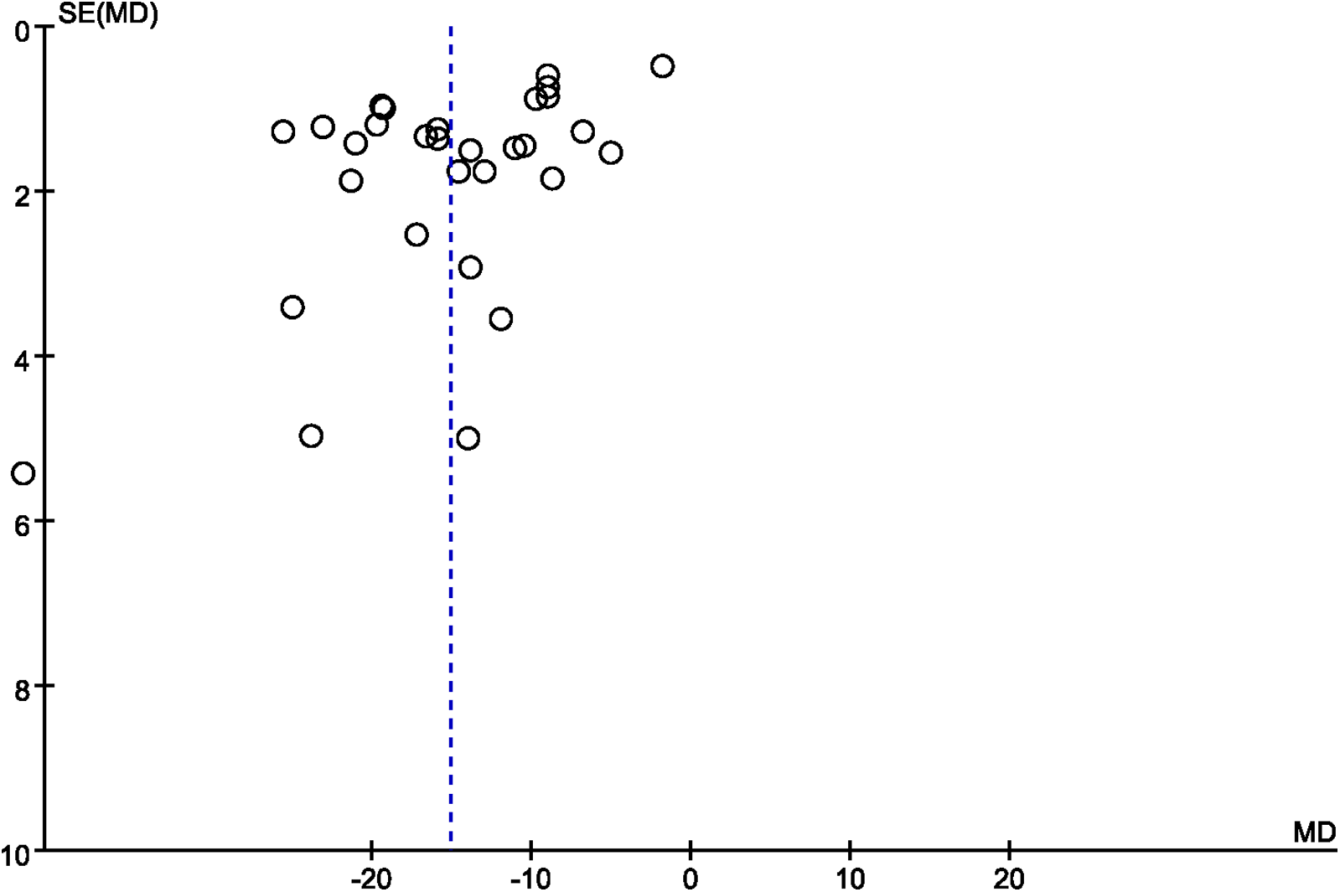
A funnel plot showing publication bias for operation time.

**Figure 12:**
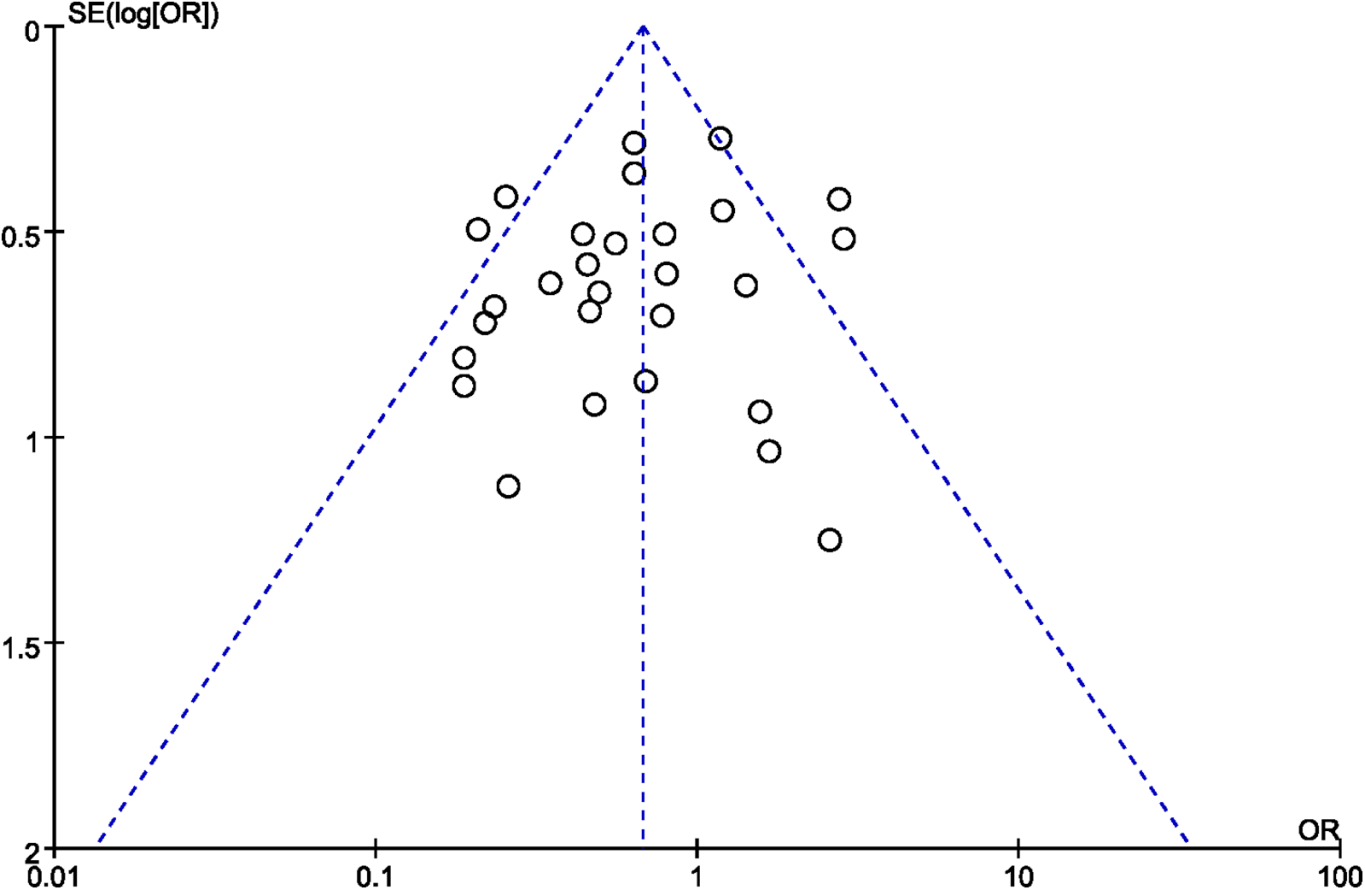
A funnel plot showing publication bias for the overall complication rate.

A funnel plot was employed to evaluate publication bias. In the studies that reported operation time and overall complication rate, the funnel plot displayed asymmetry (Figures 11, 12, respectively), indicating a possible occurrence of publication bias. Furthermore, we detected publication bias in bone cement dose, radiation dose, bone cement leakage rate, and refracture rate (see Supplementary File 2, Figures S9–S13), which indicated a possible occurrence of publication bias.

## Discussion

The aging global population has led to a rising incidence of OVCFs, particularly in regions with advanced demographic aging such as North America, Europe, and East Asia (49). Individuals aged 65 and older are disproportionately affected, with peak prevalence observed in those over 80 years (50). This age-related susceptibility is driven by progressive bone density loss, which is further exacerbated in postmenopausal women due to estrogen deficiency (51, 52). While men exhibit lower overall incidence, aging males, particularly those undergoing androgen deprivation therapy for prostate cancer, face increasing OVCF risks (53). Dual-energy x-ray absorptiometry-measured bone mineral density remains the gold standard for diagnosing osteoporosis [WHO/International Society for Clinical Densitometry (ISCD) criteria: T-score ≤ −2.5 at lumbar spine or femur] and stratifying OVCF risks (54). Although preoperative BMD assessment is not universally mandated, its integration could refine patient selection for PKP, especially in borderline cases or younger populations with secondary osteoporosis (55). In accordance with the DGOU (German Society for Orthopaedics and Trauma) classification system, the inclusion criteria prioritized patients with Type 4–5 OVCFs—characterized by persistent pain (>3 weeks) unresponsive to conservative therapy, vertebral height loss >30%, and/or dynamic instability on imaging (56). These criteria align with recommendations for PKP—a minimally invasive procedure involving vertebral stabilization via polymethylmethacrylate cement injection, aiming to stabilize fractures and restore vertebral height (57). However, controversy persists regarding unilateral vs. bilateral approaches; while unilateral PKP may reduce surgical trauma and complication risks (58), bilateral PKP is advocated for superior fracture stability and pain relief (59). This study systematically compares both techniques to establish evidence-based selection criteria tailored to patient-specific factors.

Our meta-analysis pooled data from 35 RCTs involving 3,362 participants. Unilateral and bilateral PKP demonstrated comparable effectiveness in pain relief, functional improvement, and quality of life enhancement during long-term follow-up. Short-term outcomes at 6 months favored bilateral PKP, with significant differences in ODI scores (*P* = 0.04) and Cobb angle correction (*P* < 0.00001), potentially attributable to its more symmetrical cement distribution pattern (43). However, long-term follow-up results indicate that the differences in pain relief and functional improvement between the two methods are not statistically significant. Moreover, we also found that there was no significant difference between unilateral PKP and bilateral PKP in the Cobb angle and vertebral height, suggesting that unilateral PKP is equivalent to bilateral PKP in terms of long-term efficacy. While Chen et al. (60) reported superior final pain relief with unilateral PKP in their 17-RCT analysis (*P* = 0.006), our larger sample (35 RCTs) and GRADE-evaluated evidence found equivalent long-term efficacy between the approaches. This discrepancy may reflect our inclusion of trials with standardized outcome measurement protocols and extended follow-up durations.

While unilateral and bilateral PKP demonstrated equivalent long-term outcomes for pain (VAS), function (ODI), and vertebral alignment (Cobb angle), unilateral PKP exhibited superior perioperative performance. This meta-analysis found that unilateral PKP had a shorter operation time compared to bilateral PKP (*P* < 0.00001). The surgical duration for unilateral PKP is typically shorter as it requires a procedure on only one side of the vertebra. In contrast, bilateral PKP requires procedures on both sides of the vertebra, thereby increasing the number of punctures and consequently the surgical duration and the degree of surgical trauma (61). Chen et al. (62) conducted a meta-analysis of seven studies (six were level-II evidence and one was level-III evidence), demonstrating that unilateral PKP was associated with significantly shorter operative time compared to bilateral PKP surgery. This is consistent with our findings. A meta-analysis revealed that the unilateral approach was associated with a higher mean x-ray exposure frequency than the bilateral approach (*P* < 0.05) (63). Conversely, Yin et al. (64) analyzed 10 studies and found that unilateral PKP patients received lower mean radiation doses than bilateral PKP patients—a conclusion concordant with our results. A pooled analysis suggests both unilateral and bilateral PKP represent viable options for patients with OVCF, with comparable long-term follow-up imaging outcomes and quality of life measures (65). The unilateral approach requires unilateral vertebral bone cement injection. This technique confines cement distribution to one side of the vertebral body, potentially resulting in non-uniform cement dispersion (66). In contrast, the bilateral approach employs cement injection through both pedicles. This dual-pathway administration enables more symmetrical cement distribution within the vertebral body due to bilateral reinforcement and support (67).

Unilateral PKP not only has advantages in terms of surgical duration and radiation dosage but also demonstrates a slight edge in terms of safety, with a lower incidence of complications. Furthermore, the rate of bone cement leakage in unilateral PKP is notably lower, likely due to the reduced number of punctures and the shortened operating time in the unilateral approach, thereby diminishing the risk associated with the procedure (61, 66). The primary complications encompass adjacent nerve damage, vertebral fractures, and instances of bone cement leakage. Previous studies have indicated that there is no significant difference in the risk of cement leakage (*P* > 0.05, RR = 0.86: 0.36–2.06) and postoperative adjacent-level fractures (*P* > 0.05, RR = 0.91: 0.25–3.26) between unilateral PKP and bilateral PKP (66). In addition, another research study has demonstrated that there are no differences in cement leakage and adjacent vertebral fractures (*P* = 0.06 and *P* = 0.97, respectively) (68). Despite the concurrence of our findings with prior research regarding the incidence of adjacent vertebral fractures, our study reveals a divergence in the occurrence of bone cement leakage. Analyzing the previous study revealed that fewer than 15 studies were included, and only English-language studies were considered, introducing a language bias that could have inflated the effect sizes. Conversely, our study incorporates 35 RCTs, significantly augmenting the sample size, and includes a diverse array of linguistic sources, thereby mitigating such bias. Consequently, this enhances the generalizability of the research outcomes, rendering them more credible. Furthermore, our study demonstrated that unilateral PKP involves a significantly smaller volume of bone cement compared to bilateral PKP, as the bilateral approach requires simultaneous cement injection through bilateral pedicles into the vertebral body, thereby increasing cement volume and ensuring more homogeneous distribution within the vertebral structure. The biomechanical properties of PMMA cement distribution may play a pivotal role in pain alleviation as appropriate interdigitation with the trabecular bone reduces micromotion at fracture sites, thereby diminishing adverse stimuli (69). Research indicates a positive correlation between cement injection volume and analgesic efficacy in patients undergoing PKP, with increased cement volume enhancing fracture vertebral stability and consequently reducing pain (70). However, while this achieves superior stabilization of fractured segments, the approach elevates the risk of cement leakage and may induce excessive vertebral stiffness due to oversized cement volumes, potentially leading to asymmetric stress distribution and an increased risk of adjacent vertebral fractures (63). Future investigations should focus on quantitative analysis of cement dispersion patterns (e.g., computed tomography-based three-dimensional reconstruction) to optimize injection protocols, enabling more uniform cement distribution, effective restoration of vertebral height and structural integrity, and demonstration of superior long-term therapeutic outcomes.

However, the significant heterogeneity observed in operative time (*I*^2^ = 97%), cement volume (*I*^2^ = 97%), and radiation dose (*I*^2^ = 94%) likely stems from multiple interacting factors. Differences in surgical protocols across institutions (unilateral PKP via extrapedicular vs. transpedicular approaches) and operator experience levels may substantially influence time metrics. Unreported variations in vertebral collapse severity (DGOU classification subtypes) and bone quality (T-score stratification beyond the −2.5 threshold) could modulate cement volume requirements. Disparate definitions of “operative time” (skin incision-to-closure vs. fluoroscopy time inclusion) and cement quantification methods (volumetric CT vs. intraoperative syringe measurement) were identified as potential contributors. While sensitivity analyses failed to isolate dominant sources, this likely reflects the cumulative effect of minor variations across studies rather than a single explanatory factor. Future meta analyses for PKP surgery should stratify studies by surgical technique standardization to mitigate such heterogeneity.

Regarding balanced decision-making between unilateral and bilateral PKP, clinicians should prefer unilateral PKP for elderly patients (≥80 years) with cardiopulmonary comorbidities, or those requiring urgent mobilization (e.g., dementia risk mitigation), as the shorter operative time and lower cement volume align with frailty-safety paradigms. Furthermore, bilateral PKP should be considered for younger patients (<65 years) with severe vertebral collapse (>40% height loss) or dynamic instability, where cement symmetry may delay adjacent fractures. Unilateral PKP's radiation dose advantage makes it preferable in low-resource settings with limited fluoroscopy shielding. Conversely, acute pain control of bilateral PKP may be utilized to reduce postoperative opioid dependence. Finally, developing risk calculators that integrate evidence-based pathways such as fracture morphology (DGOU classification), bone quality (T-score trajectory), and cement viscosity thresholds (high-viscosity PMMA reduces leakage) will empower surgeons to tailor approaches without compromising safety-efficacy balance.

### Current limitation

This study is not without its limitations. To begin with, despite the inclusion of 35 RCTs, the statistical power of several studies might be compromised due to their relatively small sample sizes (fewer than 50 participants). Second, the heterogeneity across these studies is notably high, with disparities in surgical techniques, types of bone cement, and baseline patient characteristics, all of which could potentially influence the consistency and comparability of the results. Third, the limited duration of follow-up in some studies underscores the need for more extensive long-term efficacy data. Finally, while the focus of this research has been principally on primary outcome measures, secondary outcomes such as patient satisfaction and improvements in quality of life may not have been adequately assessed. In addition, there remains a need to explore the distribution of bone cement and biomechanical aspects in unilateral vs. bilateral PKP procedures to better inform clinical practice.

## Conclusion

In conclusion, this meta-analysis provides valuable insights into the comparative effectiveness of unilateral vs. bilateral PKP for the treatment of OVCFs. While bilateral PKP generally offers superior outcomes, the increased risk of complications necessitates careful consideration. Future research should aim to address the identified limitations, particularly in terms of inclusivity and long-term follow-up, to provide a more comprehensive understanding of these treatment options.

## Data Availability

The original contributions presented in the study are included in the article/Supplementary Material, further inquiries can be directed to the corresponding authors.

## Supplementary material

Supplementary File 1 Literature retrieval strategy.

Supplementary File 2 Secondary meta-analysis results.

## References

[1] Wani IM Arora S. Computer-aided diagnosis systems for osteoporosis detection: a comprehensive survey. Med Biol Eng Comput. (2020) 58(9):1873–917. 10.1007/s11517-020-02171-3 32583141

[2] Capdevila-Reniu A Navarro-López M López-Soto A. Osteoporotic vertebral fractures: a diagnostic challenge in the 21(st) century. Rev Clin Esp (Barc). (2021) 221(2):118–24. 10.1016/j.rce.2019.09.006 33998487

[3] Adejuyigbe B Kallini J Chiou D Kallini JR. Osteoporosis: molecular pathology, diagnostics, and therapeutics. Int J Mol Sci. (2023) 24(19):14583. 10.3390/ijms241914583 37834025 PMC10572718

[4] Patel D Liu J Ebraheim NA. Managements of osteoporotic vertebral compression fractures: a narrative review. World J Orthop. (2022) 13(6):564–73. 10.5312/wjo.v13.i6.564 35949707 PMC9244957

[5] Keshavarzi F Arazpour M. Effect of spinal orthoses on osteoporotic elderly patients kyphosis, back muscles strength, balance and osteoporotic vertebral fractures: (A systematic review and meta-analysis). J Rehabil Assist Technol Eng. (2024) 11:20556683241268605. 10.1177/20556683241268605 39211735 PMC11359449

[6] Prost S Pesenti S Fuentes S Tropiano P Blondel B. Treatment of osteoporotic vertebral fractures. Orthop Traumatol Surg Res. (2021) 107(1s):102779. 10.1016/j.otsr.2020.102779 33321233

[7] Lu Q Liu C Wang D Liu H Yang H Yang L. Biomechanical evaluation of calcium phosphate-based nanocomposite versus polymethylmethacrylate cement for percutaneous kyphoplasty. Spine J. (2019) 19(11):1871–84. 10.1016/j.spinee.2019.06.007 31202837

[8] Zhu J Zhang K Luo K Qiu Z Yang S Cui F Mineralized collagen modified polymethyl methacrylate bone cement for osteoporotic compression vertebral fracture at 1-year follow-up. Spine (Phila Pa 1976). (2019) 44(12):827–38. 10.1097/BRS.0000000000002971 30601358

[9] Zhu D Hu JN Wang L Cui W Zhu JC Ma S A modified unilateral extrapedicular approach applied to percutaneous kyphoplasty to treat lumbar osteoporotic vertebral compression fracture: a retrospective analysis. Pain Physician. (2023) 26(3):E191–201. 10.36076/ppj.2023.26.E191 37192242

[10] Sun Y Li X Ma S Chong H Cai TC Li KM Comparison of the efficacy and safety of unilateral and bilateral approach kyphoplasty in the treatment of osteoporotic vertebral compression fractures: a meta-analysis. Jt Dis Relat Surg. (2024) 35(3):491–503. 10.52312/jdrs.2024.1701 39189557 PMC11411882

[11] Cao DH Gu WB Zhao HY Hu JL Yuan HF. Advantages of unilateral percutaneous kyphoplasty for osteoporotic vertebral compression fractures-a systematic review and meta-analysis. Arch Osteoporos. (2024) 19(1):38. 10.1007/s11657-024-01400-8 38750277

[12] Li T Yan J Liu X Hu J Wang F. Efficacy and safety of conservative treatment compared with surgical treatment for thoracolumbar fracture with score 4 thoracolumbar injury classification and severity (TLICS): a systematic review and meta-analysis. Clin Spine Surg. (2023) 37(5):230–41. 10.1097/BSD.0000000000001503 37448163 PMC11142650

[13] Li T Yan J Hu J Liu X Wang F. Efficacy and safety of electroacupuncture for carpal tunnel syndrome (CTS): a systematic review and meta-analysis of randomized controlled trials. Front Surg. (2022) 9:952361. 10.3389/fsurg.2022.952361 36211261 PMC9539120

[14] Ceng Y Xu J. Staged injection of bone cement in percutaneous vertebroplasty. Chin J Tissue Eng Res. (2013) 17(12):2116–22. 10.3969/j.issn.2095-4344.2013.12.004

[15] Chen C Bian J Zhang W Zhang W Zhao C Wei H. Unilateral versus bilateral vertebroplasty for severe osteoporotic vertebral compression fractures. J Spinal Disord Tech. (2014) 27(8):E301–4. 10.1097/BSD.0000000000000118 24901876

[16] Chen C Chen L Gu Y Xu Y Liu Y Bai X Kyphoplasty for chronic painful osteoporotic vertebral compression fractures via unipedicular versus bipedicular approachment: a comparative study in early stage. Injury. (2010) 41(4):356–9. 10.1016/j.injury.2009.09.021 19818957

[17] Chen L Yang H Tang T. Unilateral versus bilateral balloon kyphoplasty for multilevel osteoporotic vertebral compression fractures: a prospective study. Spine. (2011) 36(7):534–40. 10.1097/BRS.0b013e3181f99d70 21242864

[18] Cheng Y Liu Y. Percutaneous curved vertebroplasty in the treatment of thoracolumbar osteoporotic vertebral compression fractures. J Int Med Res. (2019) 47(6):2424–33. 10.1177/0300060519836917 31007103 PMC6567733

[19] Feng Y Li F Qin F Chen W Zhang S Xie C Efficacy and safety of percutaneous vertebroplasty via unilateral pedicle approach for osteoporotic vertebral burst fracture. Hainan Med J. (2023) 34(20):2941–5. 10.3969/j.issn.1003-6350.2023.20.012

[20] Geng Z Zhou Q Shang G Ji Y Kou H Liu H. Short-term efficacy of the percutaneous vertebroplasty using a curved versus straight vertebroplasty needle in osteoporotic vertebral compression fractures. Orthopedics. (2021) 44(1):e131–8. 10.3928/01477447-20201012-03 33141228

[21] Huang S Ni F Pan B. Comparison of the short-term efficacy of PKP through unilateral or bilateral approaches in the treatment of thoracolumbar vertebral compression fractures. Anhui Med Pharmaceutical J. (2021) 25(9):1732–5. 10.3969/j.issn.1009-6469.2021.09.008

[22] Li L Wang M Ma H Zhou X Zhou J Chen Y Comparison of unilateral and bilateral penetrating kyphoplasty for osteoporotic centrum compression fractures. Orthop J China. (2014) 22(8):678–82. 10.3977/j.issn.1005-8478.2014.08.02

[23] Li Q Lu M Zheng C. Unilateral versus bilateral percutaneous kyphoplasty for the treatment of osteoportic compression fractures: a prospective case control study. Med J Wuhan Univ. (2012) 33(4):567–70. 10.14188/j.1671-8852.2012.04.024

[24] Liu C Hu Y Wang G Tang Y Wang X Hou H. Curative effects of unipedicular and bipedicular vertebroplasty in treating osteoporotic vertebral compression fractures in the elderly population. J. Xi'an Jiaotong Univ (Medical Science). (2015) 36(6):857–61. 10.7652/jdyxb201506029

[25] Liu MX Xia L Zhong J Dou NN Li B. Is it necessary to approach the compressed vertebra bilaterally during the process of PKP? J Spinal Cord Med. (2020) 43(2):201–5. 10.1080/10790268.2018.1451238 30388938 PMC7054905

[26] Lu J Huang L Chen W Luo Z Yang H Liu T. Bilateral percutaneous kyphoplasty achieves more satisfactory outcomes compared to unilateral percutaneous kyphoplasty in osteoporotic vertebral compression fractures: a comprehensive comparative study. J Back Musculoskelet Rehabil. (2023) 36(1):97–105. 10.3233/BMR-210225 35938239

[27] Lu Z Sun T Zhang J Zhang W Yang M Li Z. Kyphoplasty via different approaches for osteoporotic vertebral compression fractures. Chin J Tissue Eng Res. (2023) 27(36):5834–9. 10.12307/2023.792

[28] Mu Z Shao H. Influence of surgical approach on postoperative complications of osteoporotic thoracolumbar fracture and analysis of risk factors for postoperative refracture. Shanxi Medical Journal. (2022) 51(7):848–55. 10.3969/j.issn.1000-7377.2022.07.019

[29] Rebolledo BJ Gladnick BP Unnanuntana A Nguyen JT Kepler CK Lane JM. Comparison of unipedicular and bipedicular balloon kyphoplasty for the treatment of osteoporotic vertebral compression fractures: a prospective randomised study. Bone Joint J. (2013) 95-b(3):401–6. 10.1302/0301-620X.95B3.29819 23450028

[30] Shi X Li P Li J Bao C Xiang J Lu Y. Comparative evaluation of an innovative deflectable percutaneous kyphoplasty versus conventional bilateral percutaneous kyphoplasty for osteoporotic vertebral compression fractures: a prospective, randomized and controlled trial. Spine J. (2023) 23(4):585–98. 10.1016/j.spinee.2022.12.012 36563860

[31] Tan H Meng Z Huang T Li J Wang T Fu G. Comparison of clinical efficacy of unilateral and bilateral PKP in the treatment of osteoporotic vertebral compression fractures. Orthop J China. (2017) 25(16):1511–4. 10.3977/j.issn.1005-8478.2017.16.15

[32] Tang J Guo WC Hu JF Yu L. Unilateral and bilateral percutaneous kyphoplasty for thoracolumbar osteoporotic compression fractures. J Coll Phys Surg Pakistan. (2019) 29(10):946–50. 10.29271/jcpsp.2019.10.946 31564267

[33] Xiong XM Sun YL Song SM Yang MY Zhou J Wan D Efficacy of unilateral transverse process-pedicle and bilateral puncture techniques in percutaneous kyphoplasty for Kummell disease. Exp Ther Med. (2019) 18(5):3615–21. 10.3892/etm.2019.7980 31602238 PMC6777381

[34] Xu D Ruan C Wang Y Hu X Ma W. Comparison of the clinical effect of unilateral transverse process extrapedicular and bilateral transpedicular percutaneous kyphoplasty for thoracolumbar osteoporotic vertebral compression fracture. Front Surg. (2024) 11:1395289. 10.3389/fsurg.2024.1395289 39092152 PMC11291213

[35] Xue W. Comparison of the effect of unilateral and bilateral percutaneous puncture vertebroplasty on osteoporotic thoracolumbar vertebral compression fractures. J Xinxiang Med Univ. (2017) 34(1):69–71. 10.7683/xxyxyxb.2017.01.020

[36] Yan L He B Guo H Liu T Hao D. The prospective self-controlled study of unilateral transverse process-pedicle and bilateral puncture techniques in percutaneous kyphoplasty. Osteoporos Int. (2016) 27(5):1849–55. 10.1007/s00198-015-3430-5 26608054

[37] Yan L Jiang R He B Liu T Hao D. A comparison between unilateral transverse process-pedicle and bilateral puncture techniques in percutaneous kyphoplasty. Spine. (2014) 39(26 Spec No.):B19–26. 10.1097/BRS.0000000000000493 25504098

[38] Yang A Zou J Lu Q He C. Comparison of high viscosity cement PVP for treatment of osteoporotic vertebral compression fractures by unilateral and bilateral approach. J Pract Med. (2018) 34(14):2377–80. 10.3969/j.issn.1006-5725.2018.14.024

[39] Yin F Sun Z Song S Wei X Liu X Yin Q A comparative study on treatment of mid-thoracic osteoporotic vertebral compression fracture using percutaneous kyphoplasty with unilateral and bilateral approaches. Chin J Reparative Reconstr Surg. (2016) 30(1):77–81. 10.7507/1002-1892.20160016 27062851

[40] Yu Q Long J Kang X. Effect analysis of unilateral and bilateral percutaneous vertebroplasty for Kummell's disease. Guizhou Med J. (2020) 44(4):574–6. 10.3969/j.issn.1000-744X.2020.04.023

[41] Zhang L Liu Z Wang J Feng X Yang J Tao Y Unipedicular versus bipedicular percutaneous vertebroplasty for osteoporotic vertebral compression fractures: a prospective randomized study. BMC Musculoskelet Disord. (2015) 16:145. 10.1186/s12891-015-0590-6 26071690 PMC4465457

[42] Zhang L Yang W Ding G Li P Xiao Z Chen Y Dispersion effect of bone cement after vertebroplasty using individualized unilateral external pedicle approach and bilateral pedicle approach. Chin J Tissue Eng Res. (2024) 29(4):800–8. 10.12307/2025.253

[43] Zhang L-G Gu X Zhang H-L Zhang Q-G Cai X-B Tao K. Unilateral or bilateral percutaneous vertebroplasty for acute osteoporotic vertebral fracture: a prospective study. J Spinal Disord Tech. (2015) 28(2):E85–8. 10.1097/BSD.0000000000000147 25099973

[44] Zhang Y Chen X Ji J Xu Z Sun H Dong L Comparison of unilateral and bilateral percutaneous kyphoplasty for bone cement distribution and clinical efficacy: an analysis using three-dimensional computed tomography images. Pain Physician. (2022) 25(6):E805–13. 36122263

[45] Zhang Y Zhu G Li S Lin Y Zeng L Zhong H Comparison of efficacy of two methods in treatment of osteoporotic vertebral compression fracture in the elderly patients. J Clin Orthop. (2020) 23(3):336–40. 10.3969/j.issn.1008-0287.2020.03.012

[46] Zhou M Li S Liu H Wang C Liang Y Sun F Effect analysis of unilateral and bilateral percutaneous balloon kyphoplasty for aged patients with thoracolumbar vertebral compression fractures. Chin J Osteoporos. (2013) 19(5):488–90. 10.3969/j.issn.1006-7108.2013.05.015

[47] Zhou R Li G Liang W Feng J. Clinical analysis of percutaneous vertebroplasty with unilateral and bilateral hydraulic delivery of high viscosity bone cement in the treatment of osteoporotic vertebral compression fractures. J Trauma Surg. (2020) 22(5):345–9. 10.3969/j.issn.1009-4237.2020.05.006

[48] Zhou X Dong G Han J Yan J. Effect of different cement injection and repair methods on percutaneous kyphoplasty for elderly patients with osteoporotic thoracolumbar vertebral compression. J Trauma Surg. (2019) 21(11):863–8. 10.3969/j.issn.1009-4237.2019.11.16

[49] Bliuc D Nguyen ND Milch VE Nguyen TV Eisman JA Center JR. Mortality risk associated with low-trauma osteoporotic fracture and subsequent fracture in men and women. Jama. (2009) 301(5):513–21. 10.1001/jama.2009.50 19190316

[50] Jordan KM Cooper C. Epidemiology of osteoporosis. Best Pract Res Clin Rheumatol. (2002) 16(5):795–806. 10.1053/berh.2002.0264 12473274

[51] Jackson RD Mysiw WJ. Insights into the epidemiology of postmenopausal osteoporosis: the Women's Health Initiative. Semin Reprod Med. (2014) 32(6):454–62. 10.1055/s-0034-1384629 25321423

[52] Cranney A Jamal SA Tsang JF Josse RG Leslie WD. Low bone mineral density and fracture burden in postmenopausal women. Can Med Assoc J. (2007) 177(6):575–80. 10.1503/cmaj.070234 17846439 PMC1963365

[53] Vilaca T Eastell R Schini M. Osteoporosis in men. Lancet Diabetes Endocrinol. (2022) 10(4):273–83. 10.1016/S2213-8587(22)00012-2 35247315

[54] El Maghraoui A Roux C. DXA Scanning in clinical practice. Qjm. (2008) 101(8):605–17. 10.1093/qjmed/hcn022 18334497

[55] Wang J Xie X Gou Y Wu Y Pu H Chen Q Forearm bone mineral density as a predictor of adjacent vertebral refracture after percutaneous kyphoplasty in patients with osteoporotic vertebral compression fracture: a retrospective analysis. J Orthop Surg Res. (2024) 19(1):788. 10.1186/s13018-024-05258-x 39581973 PMC11585950

[56] Schnake KJ Blattert TR Hahn P Franck A Hartmann F Ullrich B Classification of osteoporotic thoracolumbar spine fractures: recommendations of the spine section of the German Society for Orthopaedics and Trauma (DGOU). Global Spine J. (2018) 8(2 Suppl):46s–9. 10.1177/2192568217717972 30210960 PMC6130101

[57] Aparisi F. Vertebroplasty and kyphoplasty in vertebral osteoporotic fractures. Semin Musculoskelet Radiol. (2016) 20(4):382–91. 10.1055/s-0036-1592431 27842431

[58] Phillips FM. Minimally invasive treatments of osteoporotic vertebral compression fractures. Spine (Phila Pa 1976). (2003) 28(15 Suppl):S45–53. 10.1097/01.BRS.0000076898.37566.32 12897474

[59] Buchbinder R Osborne RH Ebeling PR Wark JD Mitchell P Wriedt C A randomized trial of vertebroplasty for painful osteoporotic vertebral fractures. N Engl J Med. (2009) 361(6):557–68. 10.1056/NEJMoa0900429 19657121

[60] Chen Y Zhang H Chen H Ou Z Fu Y Zhang J. Comparison of the effectiveness and safety of unilateral and bilateral percutaneous vertebroplasty for osteoporotic vertebral compression fractures: a protocol for systematic review and meta-analysis. Medicine (Baltimore). (2021) 100(51):e28453. 10.1097/MD.0000000000028453 34941201 PMC10545173

[61] Gangi A Guth S Imbert JP Marin H Dietemann JL. Percutaneous vertebroplasty: indications, technique, and results. Radiographics. (2003) 23(2):e10. 10.1148/rg.e10 12889460

[62] Chen X Guo W Li Q Ou Z Lao Z Liu Y Is unilateral percutaneous kyphoplasty superior to bilateral percutaneous kyphoplasty for osteoporotic vertebral compression fractures? Evidence from a systematic review of discordant meta-analyses. Pain Physician. (2018) 21(4):327–36. 30045590

[63] Cheng X Long HQ Xu JH Huang YL Li FB. Comparison of unilateral versus bilateral percutaneous kyphoplasty for the treatment of patients with osteoporosis vertebral compression fracture (OVCF): a systematic review and meta-analysis. Eur Spine J. (2016) 25(11):3439–49. 10.1007/s00586-016-4395-6 26814475

[64] Yin P Ji Q Wang Y Liu Y Wu Y Yu Y Percutaneous kyphoplasty for osteoporotic vertebral compression fractures via unilateral versus bilateral approach: a meta-analysis. J Clin Neurosci. (2019) 59:146–54. 10.1016/j.jocn.2018.10.112 30414813

[65] Tan G Li F Zhou D Cai X Huang Y Liu F. Unilateral versus bilateral percutaneous balloon kyphoplasty for osteoporotic vertebral compression fractures: a systematic review of overlapping meta-analyses. Medicine (Baltimore). (2018) 97(33):e11968. 10.1097/MD.0000000000011968 30113502 PMC6112965

[66] Chen H Tang P Zhao Y Gao Y Wang Y. Unilateral versus bilateral balloon kyphoplasty in the treatment of osteoporotic vertebral compression fractures. Orthopedics. (2014) 37(9):e828–35. 10.3928/01477447-20140825-61 25350627

[67] Wang S Xu H Ni W Huang Q Wang X. Unilateral versus bilateral balloon kyphoplasty in treatment of osteoporotic vertebral compression fractures: a randomized controlled trial protocol. Medicine (Baltimore). (2020) 99(25):e20524. 10.1097/MD.0000000000020524 32569172 PMC7310769

[68] Feng H Huang P Zhang X Zheng G Wang Y. Unilateral versus bilateral percutaneous kyphoplasty for osteoporotic vertebral compression fractures: a systematic review and meta-analysis of RCTs. J Orthop Res. (2015) 33(11):1713–23. 10.1002/jor.22957 26123667

[69] Morimoto T Toda Y Hakozaki M Paholpak P Watanabe K Kato K A new era in the management of spinal metastasis. Front Oncol. (2024) 14:1374915. 10.3389/fonc.2024.1374915 38694784 PMC11062132

[70] Wang M Zhang L Fu Z Wang H Wu Y. Selections of bone cement viscosity and volume in percutaneous vertebroplasty: a retrospective cohort study. World Neurosurg. (2021) 150:e218–27. 10.1016/j.wneu.2021.02.133 33727205

