# Supplementary material for: In-depth meta-analysis: unilateral PKP demonstrates significant advantages in treating osteoporotic vertebral compression fractures—an expanded RCT study with GRADE scoring
Source: Front Surg. 2025 May 16;12:1591686. doi: 10.3389/fsurg.2025.1591686 (PMC12123880; doi:10.3389/fsurg.2025.1591686)
Supplement: Supplementary file 1 [file Table1.docx]

Table 1. Basic characteristics of the included literature

| Name | Year | Number of persons (I/C) | Age(I/C) | Intervention group | Control group | Vertebral fractures (I/C) | Study duration（m） | Outcome |
| --- | --- | --- | --- | --- | --- | --- | --- | --- |
| Ceng YW | 2013 | 12/14 | NA | Unilateral bone cement | Bilateral bone cement | NA | 6~12（9） | ②⑧ |
| Chen CM | 2010 | 33/25 | 67.73/68.52 | Unilateral bone cement | Bilateral bone cement | NA | NA | ①②⑧⑨ |
| Chen CM | 2014 | 20/19 | 69.43/68.66 | Unilateral bone cement | Bilateral bone cement | NA | NA | ①②⑨ |
| Chen L | 2011 | 24/25 | 70.4/72.4 | Unilateral bone cement | Bilateral bone cement | Lumbar 26 (I)/23(C); Thoracic 29(I)/36(C) | 31.8/35.2 | ④⑤⑥⑦ |
| Cheng YH | 2019 | 26/22 | 68.9/69.8 | Unilateral bone cement | Bilateral bone cement | Lumbar 14 (I)/16(C); Thoracic 16(I)/16(C) | 3 | ①②③⑧ |
| Feng YH | 2023 | 50/50 | 63.98/63.87 | Unilateral bone cement | Bilateral bone cement | Lumbar 22 (I)/20(C); Thoracic 28(I)/30(C) | NA | ①②③⑦⑧⑨ |
| Geng ZH | 2021 | 40/31 | 70.6/70.4 | Unilateral bone cement | Bilateral bone cement | Lumbar 27 (I)/19(C); Thoracic 13(I)/12(C) | NA | ①②⑦⑧⑨ |
| Huang SC | 2021 | 46/46 | 72.05/71.72 | Unilateral bone cement | Bilateral bone cement | NA | NA | ①②③④⑤⑧ |
| Li L | 2014 | 38/37 | 71.13/67.65 | Unilateral bone cement | Bilateral bone cement | Lumbar 24 (I)/20(C); Thoracic 18(I)/20 (C) | 24 | ①②③④⑤⑦⑧⑩ |
| Li Q | 2012 | 50/41 | 73.1/70.8 | Unilateral bone cement | Bilateral bone cement | NA | 12~36 | ①②③⑧ |
| Liu CL | 2015 | 48/50 | 70.14/70.52 | Unilateral bone cement | Bilateral bone cement | NA | 15.81/15.42 | ①②⑧⑨ |
| Liu MX | 2018 | 42/43 | 67.7/70.5 | Unilateral bone cement | Bilateral bone cement | NA | 12 | ①②⑦⑧⑨ |
| Lu JH | 2022 | 37/42 | 67.4/70.3 | Unilateral bone cement | Bilateral bone cement | Lumbar 20 (I)/21(C); Thoracic 17(I)/21(C) | 24 | ①②③④⑤⑥⑦ |
| Lu ZH | 2022 | 175/208 | 72.3/74.1 | Unilateral bone cement | Bilateral bone cement | Lumbar 79 (I)/93(C); Thoracic 96(I)/115(C) | 28~98（43.3） | ①②③⑦⑧⑨⑩ |
| Mu ZZ | 2022 | 80/73 | 62.13/63.51 | Unilateral bone cement | Bilateral bone cement | Lumbar 44 (I)/43(C); Thoracic 36(I)/36(C) | 29.92/30.28 |  |
| Rebollede BJ | 2013 | 23/21 | 78.7/79.3 | Unilateral bone cement | Bilateral bone cement | Lumbar 7 (I)/2(C); Thoracic 21(I)/26(C) | NA | ①②⑦ |
| Shi X | 2022 | 40/45 | 71.38/70.64 | Unilateral bone cement | Bilateral bone cement | NA | NA | ①②③④⑧⑨ |
| Tan HT | 2018 | 66/66 | 69.3/68.4 | Unilateral bone cement | Bilateral bone cement | Lumbar 35 (I)/38(C); Thoracic 44(I)/48(C) | 12 | ①③④⑦⑧ |
| Tang J | 2019 | 83/95 | 72.3/73.9 | Unilateral bone cement | Bilateral bone cement | Lumbar 42 (I)/51(C); Thoracic 41(I)/44(C) | 9.3/8.5 | ①②③④⑤⑦⑧⑨ |
| Xiong XM | 2019 | 38/25 | 69.7/69.4 | Unilateral bone cement | Bilateral bone cement | Lumbar 26 (I)/14(C); Thoracic 12(I)/11(C) | 12 | ①②③⑤⑦⑧⑨ |
| Xu DL | 2024 | 62/74 | 69.4/68.8 | Unilateral bone cement | Bilateral bone cement | Lumbar 36 (I)/39(C); Thoracic 26(I)/35(C) | 12 | ①②③⑦⑧⑨ |
| Xue W | 2017 | 38/38 | 67.89/69.37 | Unilateral bone cement | Bilateral bone cement | Lumbar 23 (I)/22(C); Thoracic 15(I)/16(C) | 12 | ①②⑤⑦⑧⑨ |
| Yan L | 2015 | 55/53 | 68.9 | Unilateral bone cement | Bilateral bone cement | NA | 12 | ①②⑦⑧ |
| Yan L | 2014 | 158/151 | 71.9/71.1 | Unilateral bone cement | Bilateral bone cement | NA | 12~28（16.8） | ①②⑦ |
| Yang AF | 2018 | 45/46 | 75.2/76.1 | Unilateral bone cement | Bilateral bone cement | Lumbar 20 (I)/21(C); Thoracic 25(I)/25(C) | 6~12 | ①③⑦⑧⑨⑩ |
| Yin F | 2016 | 11/11 | 81.3/82.5 | Unilateral bone cement | Bilateral bone cement | Thoracic 11(I)/11(C) | 13~35（15.3） | ①③⑦⑧ |
| Yu Q | 2020 | 16/16 | 68.74/70.91 | Unilateral bone cement | Bilateral bone cement | NA | 6 |  |
| Zhang L | 2015 | 24/26 | 69.2/70.5 | Unilateral bone cement | Bilateral bone cement | NA | 24 | ①②③⑧⑨ |
| Zhang LC | 2023 | 36/35 | 72.69/71.86 | Unilateral bone cement | Bilateral bone cement | Lumbar 31 (I)/30(C); Thoracic 9(I)/10(C) | NA | ①②③⑧⑨ |
| Zhang LG | 2015 | 36/32 | 70/70.7 | Unilateral bone cement | Bilateral bone cement | NA | 12 | ⑧ |
| Zhang YH | 2020 | 32/28 | NA | Unilateral bone cement | Bilateral bone cement | Lumbar 11 (I)/10(C); Thoracic 25(I)/23(C) | 6~13 | ①⑧ |
| Zhang YT | 2022 | 29/38 | 73.6/74.1 | Unilateral bone cement | Bilateral bone cement | NA | 14~27（17.1） | ①②⑧ |
| Zhou MW | 2013 | 30/37 | 67.1/67.1 | Unilateral bone cement | Bilateral bone cement | NA | 18~54（28.6） | ①②④⑤⑦⑧ |
| Zhou RL | 2020 | 59/59 | 72.3/72.3 | Unilateral bone cement | Bilateral bone cement | Lumbar 30 (I)/32(C); Thoracic 16(I)/15 (C) | NA | ①②③④⑤⑥⑦⑧⑨ |
| Zhou X | 2019 | 69/69 | 71.47/70.47 | Unilateral bone cement | Bilateral bone cement | Lumbar 14 (I)/16(C); Thoracic 12(I)/10(C) | 12 | ①②③⑦⑩ |

①: Operation time (min); ②: Cement dose (ml); ③: Radiation dose; ④: Anterior vertebral height; ⑤: Middle vertebral height; ⑥: Posterior vertebral height; ⑦: Cobb angle (o); ⑧: VAS (Visual analogue scale); ⑨: ODI (Oswestry Disability Index); ⑩: Hospital stays; : Re-fracture; : Cement leakage; : Overall complication; NA: Not available
