# Supplementary material for: In-depth meta-analysis: unilateral PKP demonstrates significant advantages in treating osteoporotic vertebral compression fractures—an expanded RCT study with GRADE scoring
Source: Front Surg. 2025 May 16;12:1591686. doi: 10.3389/fsurg.2025.1591686 (PMC12123880; doi:10.3389/fsurg.2025.1591686)
Supplement: Supplementary file 2 [file Table2.docx]

GRADE assessment of clinical outcomes

| Certainty assessment | | | | | | |  | No. of patients | |  | Effect | |  | Certainty | Importance |
| --- | --- | --- | --- | --- | --- | --- | --- | --- | --- | --- | --- | --- | --- | --- | --- |
| No. of studies | Study design | Risk of bias | Inconsistency | Indirectness | Imprecision | Other considerations |  | Unilateral PKP | Bilateral PKP |  | Relative (95% CI) | Absolute (95% CI) |  |  |  |
| OT (N=30) | RCTs | Serious ^a^ | Very serious ^c^ | No serious | Very Serious ^e^ | No |  | 1530 | 1556 |  | MD -15.09 (-17.72 to -12.46) | |  | ⨁◯◯◯Very low | Importance |
| Bone cement dose (N=27) | RCTs | Serious ^a^ | Very serious ^c^ | Not serious | No | No |  | 1355 | 1402 |  | MD -1.34 (-1.76 to -0.93) | |  | ⨁◯◯◯Very low | Importance |
| Radiation dose (N=18) | RCTs | Serious ^a^ | Very serious ^c^ | No serious | Very Serious ^e^ | No |  | 955 | 997 |  | SMD -2.14 (-2.62 to -1.67) | |  | ⨁◯◯◯Very low | Importance |
| Cobb angle (N=20) | RCTs | Serious ^a^ | Serious ^b^ | No serious | No | No |  | 1902 | 1953 |  | MD 0.05 (-0.17 to 0.28) | |  | ⨁⨁◯◯ Low | Importance |
| VAS (N=27) | RCTs | Serious ^a^ | No serious | No serious | No serious | No |  | 2559 | 2598 |  | MD 0.01 (-0.03 to 0.05) | |  | ⨁⨁⨁◯Moderate | Importance |
| ODI (N=16) | RCTs | Serious ^a^ | Serious ^b^ | No serious | No serious | No |  | 1700 | 1745 |  | MD -0.06 (-0.44 to 0.32) | |  | ⨁⨁◯◯ Low | Importance |
| Bone cement leakage (N=26) | RCTs | Serious ^a^ | No serious | No serious | No serious | No |  | 165/1330 (12.4%) | 236/1355 (17.4%) |  | OR 0.64 (0.51 to 0.80) | |  | ⨁⨁⨁◯Moderate | Importance |
| Overall complication (N=28) | RCTs | Serious ^a^ | No serious | No serious | No serious | No |  | 243/1548 (15.7%) | 334/1516 (22.0%) |  | OR 0.67 (0.56 to 0.81) | |  | ⨁⨁⨁◯Moderate | Importance |
| Anterior vertebral height (N=9) | RCTs | Serious ^a^ | No serious | No serious | Serious ^d^ | No |  | 422 | 453 |  | SMD -0.04 (-0.17 to 0.1) | |  | ⨁⨁◯◯ Low | No |
| Middle vertebral height (N=9) | RCTs | Serious ^a^ | No serious | No serious | Serious ^d^ | No |  | 392 | 405 |  | SMD -0.05 (-0.19 to 0.09) | |  | ⨁⨁◯◯ Low | No |
| Posterior vertebral height (N=3) | RCTs | Serious ^a^ | No serious | No serious | Serious ^d^ | No |  | 120 | 126 |  | SMD 0.04 (-0.21 to 0.29) | |  | ⨁⨁◯◯ Low | No |
| 6 months follow-up height (N=5) | RCTs | Serious ^a^ | No serious | No serious | Serious ^d^ | No |  | 440 | 460 |  | SMD -0.14 (-0.27 to 0.00) | |  | ⨁⨁◯◯ Low | No |
| 12 months follow-up height (N=3) | RCTs | Serious ^a^ | No serious | No serious | No serious | No |  | 142 | 129 |  | MD 0.07 (-0.33 to 0.47) | |  | ⨁⨁⨁◯Moderate | No |
| Cobb angle improvement (N=3) | RCTs | Serious ^a^ | No serious | No serious | Very Serious ^e^ | No |  | 144 | 137 |  | SMD -0.03 (-0.35 to 0.29) | |  | ⨁◯◯◯Very low | No |
| Hospital days (N=4) | RCTs | Serious ^a^ | No serious | No serious | No serious | No |  | 327 | 360 |  | MD -0.16 (-0.57 to 0.26) | |  | ⨁⨁⨁◯Moderate | No |
| Re-fracture (N=11) | RCTs | Serious ^a^ | No serious | No serious | No serious | No |  | 73/802 (9.1%) | 75/846 (8.9%) |  | OR 1.00 (0.71 to 1.40) | |  | ⨁⨁⨁◯Moderate | Importance |
| Postoperative pain (N=3) | RCTs | Serious ^a^ | No serious | No serious | No serious | No |  | 17/208 (8.2%) | 25/211 (11.8%) |  | OR 0.60 (0.31 to 1.17) | |  | ⨁⨁⨁◯Moderate | No |

Notes:

CI: confidence interval; OR: odds ratio; SMD: standardized mean difference; MD: mean difference.

Explanations:

a > 50% of trials received “High” risk of bias ratings (≥1 out of 6 dimensions in the Cochrane Risk of Bias tool);

b I^2^ between > 50% and ≤75% points in either direction;

c I^2^ >75% points in either direction;

d 95% CI of an SMD extends between > 0.2 and ≤0.5 points in either direction, 95% CI of an MD extends between > 2.0 and ≤5.0 points in either direction

e 95% CI of an SMD extends >0.5 points in either direction, 95% CI of an MD extends >5.0 points in either direction;
